# High Arctic “hotspots” for sperm whales (*Physeter macrocephalus*) off western and northern Svalbard, Norway, revealed by multi-year Passive Acoustic Monitoring (PAM)

**DOI:** 10.1038/s41598-024-56287-9

**Published:** 2024-03-09

**Authors:** Viivi Pöyhönen, Karolin Thomisch, Kit M. Kovacs, Christian Lydersen, Heidi Ahonen

**Affiliations:** 1 Norwegian Polar Institute, Fram Centre, 9296 Tromsø, Norway; 2 Ocean Acoustics Group of the Alfred Wegener Institute, Helmholtz Centre for Polar and Marine Research, 27570 Bremerhaven, Germany

**Keywords:** Marine biology, Animal migration, Biodiversity

## Abstract

Despite the well-documented, broad global distribution of sperm whales (*Physeter macrocephalus*), their distributional patterns remain poorly known in Arctic regions, where year-round monitoring is challenging. Adult male sperm whales are known to migrate seasonally between nutrient-rich high latitude waters and low latitude breeding grounds. However, knowledge is limited regarding fine-scale distribution and seasonal presence at high latitudes. To investigate the acoustic occurrence of this vocally active species in the High Arctic of the Northeast Atlantic, this study combined automated and manual click detection methods to analyze passive acoustic data collected at eight locations around the Svalbard Archipelago, Norway, between 2012 and 2021. The results revealed the presence of sperm whales at six recording sites and demonstrated sperm whale “hotspots” in ice-free areas in eastern Fram Strait along the shelf break and close to the west coast of Spitsbergen from May–January, with some variation between years and locations. Although acoustic presence decreased with increasing latitude, even the northern-most location (81° N) recorded sperm whale vocal activity between August and January. This study provides a baseline for sperm whale acoustic presence in the High Arctic, which will be essential in the context of detecting future changes and also for predicting future distribution patterns in the rapidly changing Arctic marine environment.

## Introduction

The sperm whale (*Physeter macrocephalus*) is an extremely cosmopolitan species that ranges throughout the world’s oceans from the tropics to the poles^1^. It is estimated that the global population size for this species was as high as 1,900,000 individuals prior to whaling^2^. Although there are uncertainties in current sperm whale population sizes and trends, the global estimate is approximately 840,000 individuals^2^. The species is classified as “Vulnerable” on the IUCN Red List of Threatened Species^1^. Females and calves inhabit mid-latitude regions year-round, while mature males migrate between nutrient-rich high latitude waters in summer and lower latitude breeding grounds in winter. Females live in distinct social clans with other females and their calves^3^. Male sperm whales, on the other hand, are often found alone or in loose, all-male aggregations of 10–30 individuals, often spread over a large area, with individuals some kilometers apart. However, these large clusters are uncommon, and males appear to be more solitary and less social than females^4^. It is believed that the oldest and largest males are found at the highest latitudes^5^.

Even though sperm whales are known to be seasonally resident in the Arctic, their geographic distribution at a finer scale and the timing of their arrivals and departures from the north are not well documented^5^. Sea ice is known to limit their distribution; sperm whales typically avoid sea ice covered areas^6,7^. In the North Pacific, sperm whales are found in Alaskan waters^8,9^, in the Bering Sea, the Bering Strait and the southern Chukchi Sea, up to latitudes of about 68° N^7,10^. Further north, sea ice concentrations are relatively high year-round and there are no records of sperm whales. In contrast, in the North Atlantic, the sperm whales’ range extends much further north. Studies have reported sperm whales at latitudes of 75° N in Baffin Bay in the Northwest Atlantic^6,11,12^. In the Northeast Atlantic, the sperm whale’s range extends north to the Svalbard Archipelago, with observations as far as 81° N^13–17^. This is likely explained by the inflow of warm Atlantic Water in the West Spitsbergen Current, which keeps western and northern Svalbard waters relatively ice-free for most of the year^18^. Recent studies report a possible northward shift in sperm whale distribution with more frequent observations at higher latitudes around Svalbard as the northern sea ice edge has shifted northwards in recent years^17–19^. Sperm whales have also been reported in the relatively shallow waters of the Barents Sea, east of Svalbard, and strandings have occurred on the mainland in the western Russian Arctic^20^.

The High Arctic poses logistical challenges for observation-based data collection. Traditional visual survey methods are spatially and temporally limited in this vast region, with winter darkness precluding observations in winter months entirely. In the case of sperm whales, visual surveys, even under ideal conditions, can lead to underestimation of the species’ presence due to their long absences from the surface (while diving) and their low, dispersed blows when breathing at the surface can be overlooked easily^15,21^. Satellite tracking studies enable investigation of wide-ranging cetaceans^22,23^, but involve complicated and expensive logistics. One way to overcome some of these limitations is by using Passive Acoustic Monitoring (PAM), which consists of deployment of underwater recorders to capture sounds from the surrounding environment. PAM is a cost-effective, non-invasive method that is widely used for long-term studies to assess and monitor marine biodiversity, including species distribution and behavior of acoustically active species^9,11,24^. PAM also allows year-round data collection independent of sea or weather conditions in remote areas, like the High Arctic. However, PAM requires target species to be vocally active. Sperm whales produce stereotypic broadband frequency clicks during much of the time (up to 68% of the dive cycle) when they are diving, making them ideal subjects for PAM^25–28^. They produce several types of clicks including usual clicks and buzz clicks, which are highly directional broadband signals that serve as biosonar (echolocation) signals used for navigation and foraging^25,28–30^. Two other click types, codas and slow clicks, are used for socializing and general communication. Codas are mainly produced by females. These sounds are highly individualistic and might be used as individual identification codes^29,31^. Slow clicks, or ‘clangs’, which are usually produced by males^30^, are thought to serve for long range communication^32^. Propagation range estimates for sperm whale clicks vary between 6 and 80 km in various studies depending on location, season and click type^30,33,34^.

**Figure 1:**
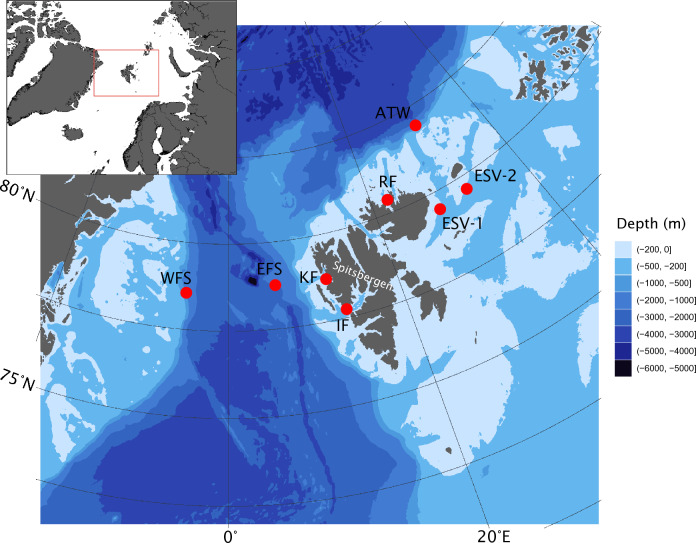
Map of mooring locations around Svalbard. Depth is given in meters. *WFS* Western Fram Strait, *EFS* Eastern Fram Strait, *KF* Kongsfjorden, *IF* Isfjorden, *RF* Rijpfjorden, *ATW* Atwain, *ESV-1* Eastern Svalbard 1, *ESV-2* Eastern Svalbard 2. Map created in R using *terra* (package version 1.7.-65; available at https://cran.r-project.org/web/packages/terra/index.html), *ggplot2* (Version 3.4.2.; available at https://cran.r-project.org/web/packages/ggplot2/index.html) and *tidyterra* (Version 0.5.2.; available at https://cran.r-project.org/web/packages/tidyterra/index.html).

Several PAM-based studies have been conducted on sperm whale populations in recent years in different parts of the world, e.g., in the Antarctic^35^, off South Africa^34^, in the Bering Sea^7^, western North Atlantic^36^, Canadian Atlantic Arctic^6^ and off Japan^37^. However, no year-round, multi-year PAM studies have been published on sperm whale spatial and temporal vocal presence in the Northeast Atlantic High Arctic. This study provides a first multi-year assessment of acoustic presence of sperm whales around the Svalbard Archipelago, Norway. Passive acoustic data from eight different locations (Fig. 1) across a 10-year cumulative time span were analyzed using a combination of customized automated and manual detection methods to obtain a spatial and temporal baseline for sperm whale acoustic presence in this region. In addition, the relationship to environmental factors such as sea ice cover and biological productivity were explored to gain further insight into the potential drivers of sperm whale distribution in the study area.

## Results

### Sperm whale detections and detector performance

**Figure 2:**
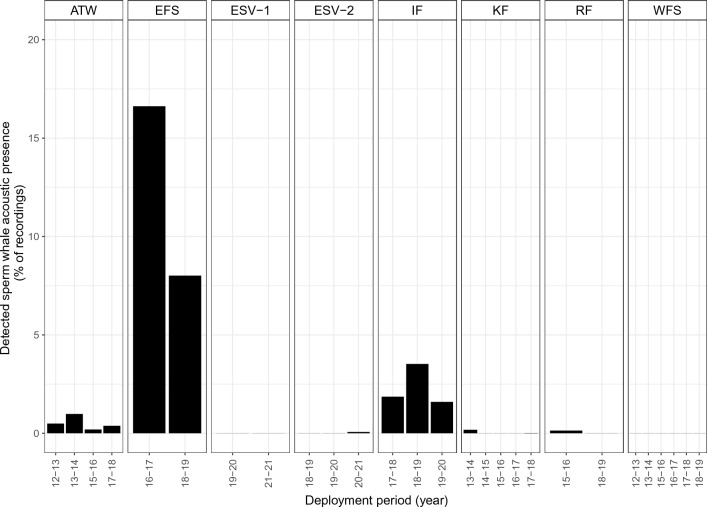
Sperm whale presence detection rate per location and recording period (years in 2000s). *WFS* Western Fram Strait, *EFS* Eastern Fram Strait, *KF* Kongsfjorden, *IF* Isfjorden, *RF* Rijpfjorden, *ATW* Atwain, *ESV-1* Eastern Svalbard 1, *ESV-2* Eastern Svalbard 2.

Sperm whale vocal presence was successfully identified in 2763 h out of a total of approximately 190,000 h, at six of the eight study locations (Fig. 2, Appendix Table A1). Most detections occurred at the Eastern Fram Strait and Isfjorden moorings, with 2125 of 17,150 h (12.3% of recordings) and 471 of 20,649 h (2.3%) containing vocal activity at these sites, respectively. At Atwain, sperm whales were detected at a low rate, with sperm whale clicks in only 142 of 32,351 h (0.4% of recordings), but they were consistently present across four deployment years. At Kongsfjorden, Rijpfjorden and in Eastern Svalbard 2, vocal activity was detected in very few recordings (< 0.1% of recordings in all cases). No sperm whales were detected at the Western Fram Strait or at the Eastern Svalbard 1 mooring during any of the deployment periods.

The automated detector was useful for identifying sperm whale presence in the vast amount of data collected by the PAM array. Periods containing ‘strong’ sperm whale vocalizations were particularly well detected (89% and 96% of hours with presence successfully detected in the detector validation datasets). For examples of detected click types and detector performance details, see Appendix Tables B1 and B2. The main sources of false positive events were identified as sea ice associated sounds (Appendix Figure C1) and self-noise from cable-strumming by the instrument or other components on the mooring (Appendix Figures C2 and C3). Occasional false positive detections were also triggered by walruses (*Odobenus rosmarus*) (Appendix Figures C4 and C5) or other odontocete sounds, such as narwhal (*Monodon monoceros*) clicks (Appendix Figure C6). The prevalence of false positive triggers varied across locations and years.

### Temporal presence

**Figure 3:**
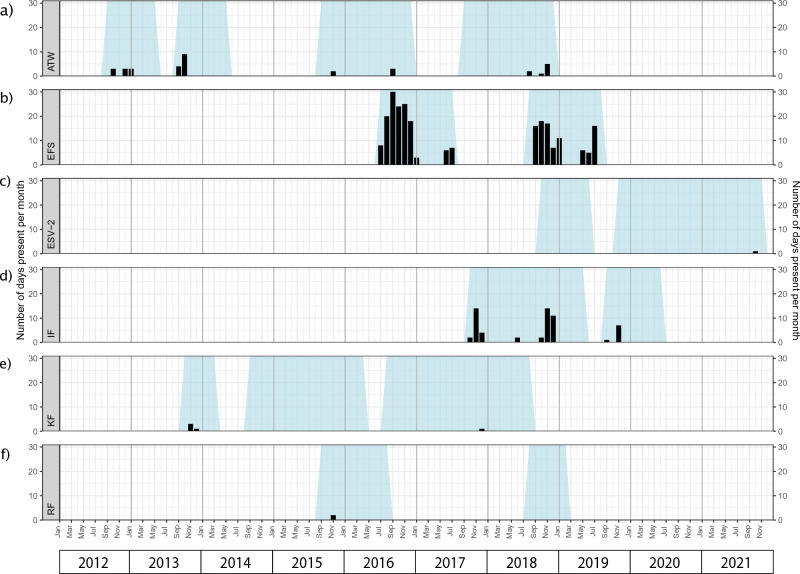
Monthly sperm whale acoustic presence at each location across all recording periods. Light blue shading: recording periods, black bars: sperm whale acoustic presence—days per month.

At the six locations that had sperm whale detections, only Eastern Fram Strait, Isfjorden and Atwain demonstrated consistent, multi-year presence across the deployment periods. Sperm whales were detected at these locations from May until January, but predominantly between September and December. The highest presence was detected at Eastern Fram Strait (Fig. 3b), where sperm whales were detected during 18 and 30 days per month in September 2016 and October 2018, respectively. The earliest spring detections at Eastern Fram Strait occurred in May 2019 and the latest seasonal durations in the north extended until January 2017 and 2019. At Isfjorden (Fig. 3d), detections occurred from June to December, but most sperm whale vocalizations occurred from October to December. Sperm whale presence peaked in November of every deployment year at Isfjorden, with 14 days (2017 and 2018) and 7 days (2019). At Atwain (Fig. 3a), sperm whale presence was detected between August and January, with a maximum of 9 days a month(October 2013). Sperm whale presence at Kongsfjorden, Rijpfjorden and Eastern Svalbard 2 was limited to only a few days over the whole study period: 2 days (Nov 2015) at Rijpfjorden, 5 days (Nov–Dec 2013 and 2017) at Kongsfjorden, and 1 day (Oct 2021) at Eastern Svalbard 2 (Fig. 3c,e,f). In February, March and April, no sperm whales were detected at any of the mooring locations in any of the study years.

**Figure 4:**
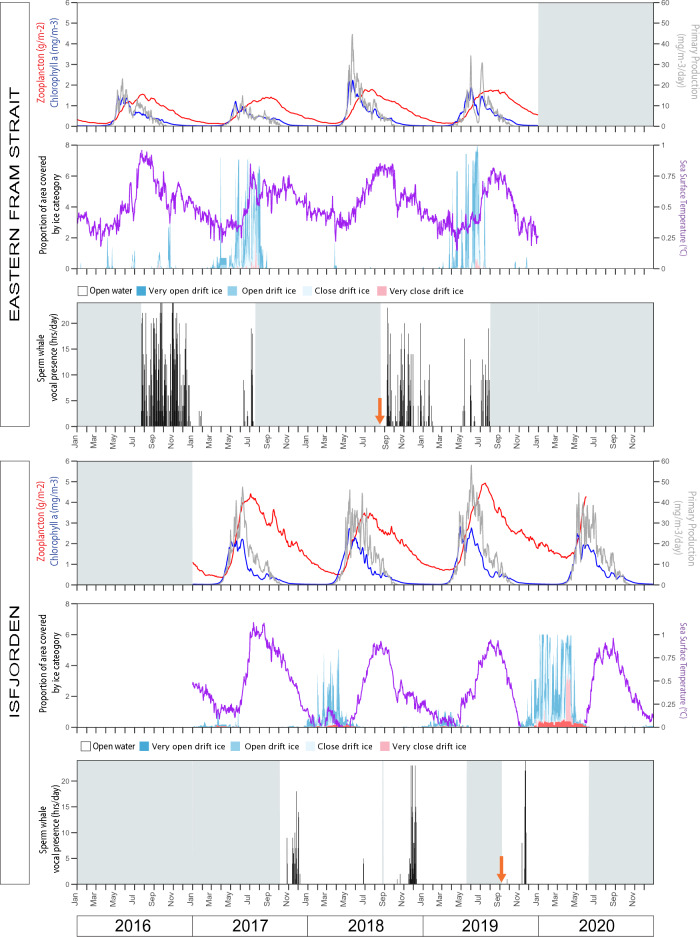
Daily mean net primary production, zooplankton and chlorophyll a concentration, daily sea ice cover, daily mean sea surface temperature and daily sperm whale acoustic presence at Eastern Fram Strait (top) and Isfjorden (bottom). Orange arrow marks the time point when the mooring was moved. Shaded (grey) areas indicate no data available.

**Figure 5:**
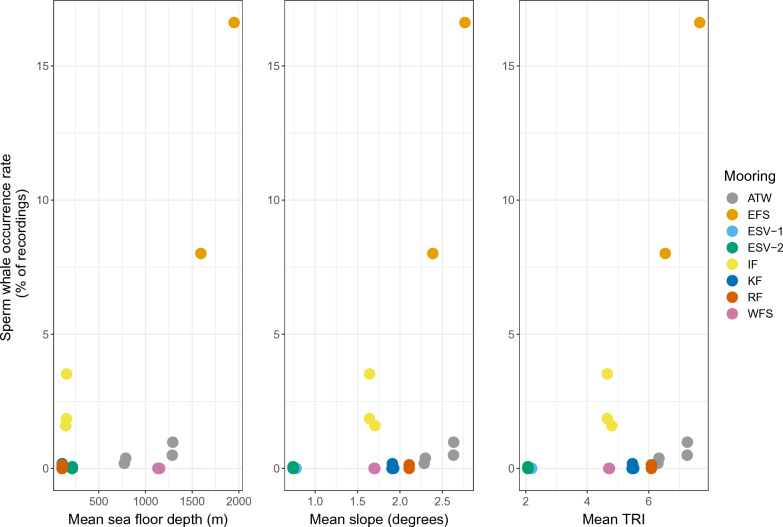
Mean depth, slope, and TRI (Terrain Ruggedness Index) at all locations (50 km radius around mooring). In cases where moorings were moved, multiple data points exist per location. *WFS* Western Fram Strait, *EFS* Eastern Fram Strait, *KF* Kongsfjorden, *IF* Isfjorden, *RF* Rijpfjorden, *ATW* Atwain, *ESV-1* Eastern Svalbard 1, *ESV-2* Eastern Svalbard 2.

Only Eastern Fram Strait and Isfjorden had enough sperm whale detections to explore statistical relationships with biological covariates, ice cover and SST (Fig. 4). The probability of sperm whale occurrence was found to increase significantly with daily mean zooplankton concentration (OR 9.36, CI_95%_ 2.42–36.17, p < 0.01) at Eastern Fram Strait, while there were no significant effects of sea ice, sea surface temperature or other biological variables. In Isfjorden, no significant effects were found for any of the environmental variables (see Appendix Figure A1). Comparing all locations, mean sea ice cover was significantly lower at times and locations where sperm whales were detected (Z = − 20.57, p < 0.005). For the rest of the locations, relationships to biological variables and SST were only examined visually (Appendix Figures A2–A7). At these locations, sperm whale activity increased after peaks in biological activity and SST, with different time lags across recording sites. The locations with the highest sperm whale detection rates were characterized by the highest sea floor mean slope and terrain heterogeneity (Fig. 5).

**Figure 6:**
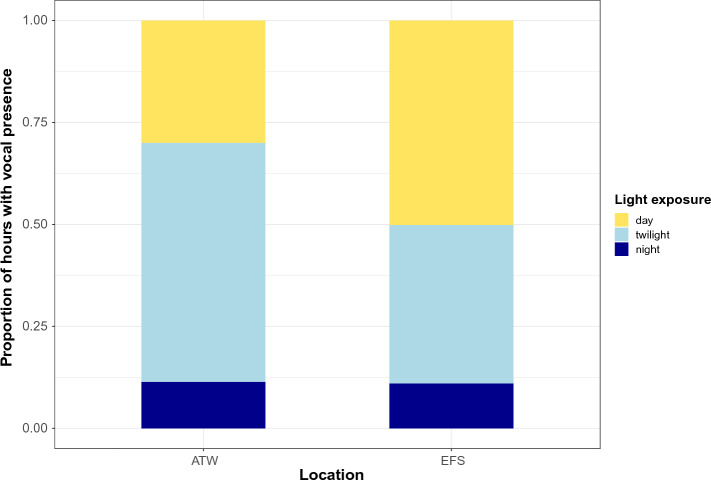
Number of recording hours with acoustic presence of sperm whales during "day-night season" according to light exposure of the hour: day (solar elevation higher than 0°), twilight (solar elevation between 0° and − 24°) or night (solar elevation below − 24°). *ATW* Atwain, *EFS* Eastern Fram Strait.

**Figure 7:**
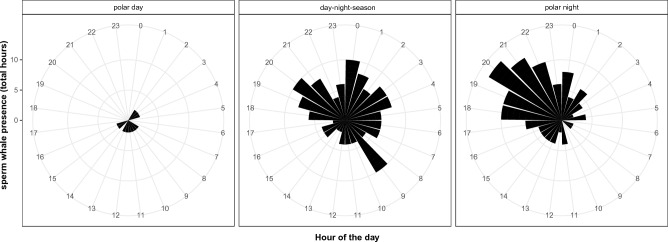
Total number of hours with acoustic presence of sperm whales according to the hour of the day under three different light regimes at Atwain.

Diurnal variation in detections during three different Arctic light regimes (seasons) were visually compared at Eastern Fram Strait, Isfjorden and Atwain. Most detections occurred during seasons with light and dark hours or continuous darkness (Polar Night). During the day-night season, most detections occurred during daylight at Eastern Fram Strait and during twilight at Atwain (Fig. 6). During the Polar Night, most detections occurred between 18:00 and 24:00 at Atwain (Fig. 7). At Isfjorden, all detections occurred during the Polar Night.

### Occurrence of vocalization types

Usual clicks were present in nearly all recordings with sperm whale detections (Appendix Figure A7). Recordings containing buzz clicks were found in similar proportions at Isfjorden and Eastern Fram Strait (15.3% and 12.85% of the total number of recordings containing sperm whale detections at each location, respectively) but much less frequently at Atwain (2.82%). Slow clicks were present most commonly in Isfjorden and least commonly at Eastern Fram Strait. Overlapping vocalizations from more than one individual occurred in more than half of the recordings with sperm whale presence. For examples of detected clicks, see spectrograms in Appendix Figures C7–C15.

## Discussion

This is the first multi-year, multi-location study of sperm whale acoustic presence in the Svalbard area. The results provide new insights into the spatial and temporal trends of sperm whale vocal presence in the High Arctic. Presence was detected at six of eight locations with two “hotspots” off Western Spitsbergen (Eastern Fram Strait and Isfjorden). Although acoustic presence decreased with increasing latitude, seasonal multi-year presence was detected even at the northernmost location in our study (Atwain—81° N), which is the northernmost record for sperm whale acoustic presence. While the automated detector was useful for screening large amounts of data, manual validation of the detector results was needed (i.e., removal of all false positive detections from the results) because of differences in false positive detection rates across locations (Appendix Table B2). Previous studies have already described the complexities of High Arctic soundscapes, characterized by a large variety of sounds of abiotic and biotic origins that exhibit strong seasonal variation^38–41^. Prevalent sounds such as sea ice or wind, as well as vocally active marine mammals, set challenges for signal detection because the likelihood of both signal masking and false detections is higher compared to many lower latitude soundscapes. The variations in detector performance across the study locations further reflect the high variability in soundscapes even within the High Arctic, highlighting the need for soundscape-specific detectors as well as the need for more annotated location-specific training data.

The Eastern Fram Strait location had the highest sperm whale occurrence in our study. While a visual survey from 1995 to 2001 did not report sperm whale sightings in the Eastern Fram Strait^42^, more recent studies from the same area do report their presence^14,16,17,19^. At Isfjorden, the overall occurrence rate, while much lower than at Eastern Fram Strait, was still higher than expected. Previous studies have reported some sightings around the mouth of this fjord^16,17,19^, but in general sperm whales are not common in the area. Sperm whales are known to avoid ice-covered areas^6,11,35^ and these two locations had the lowest overall ice cover of the eight sites during the deployment periods (Fig. 4). The Isfjorden mooring was moved after two deployment years, shifting to a site deeper into the fjord with a higher ice coverage surrounding the location. Sperm whale presence decreased following the move, compared to the first two years. Consequently, sea ice cover likely explains the total absence of sperm whale detections at the Western Fram Strait recording site (Appendix Figure A7) as the site was covered with very close drift ice most months each study year. The Western Fram Strait is a known habitat for ice-associated species such as narwhal^43^ and bowhead whales (*Balaena mysticetus*)^44^ and the former species shares a squid diet with sperm whales^45^ so lack of prey in the area is an unlikely explanation of their absence.

Similarly, the Eastern Svalbard locations had no detections, except for a single day at the Eastern Svalbard 2. These sites were also ice covered during most of the data collection periods (Appendix Figures A2 and A3). Although Rijpfjorden had longer open water periods (Appendix Figure A6), sperm whale presence was also limited to a couple of days during two recording years. This may be explained by the local bathymetry and oceanography. Sperm whales are known to forage in deep waters near shelf-edges^17,46,47^ rather than shallow fjord areas. The Rijpfjorden mooring is farther away from the shelf-edge and even though sperm whales sometimes do forage in shallower waters^48–50^, prey availability might be limited in this fjord, considering the extensive ice cover and short growing season. However, this mooring is located relatively close (< 10 km) to the mouth of the fjord, so detected vocal activity at this site might have arisen from individuals outside the fjord.

The Eastern Fram Strait moorings are located right at a shelf edge near the deepest part of Fram Strait, with the greatest sea floor depth, slope, and heterogeneity (Fig. 5). This region of the central Fram Strait is also where warm Atlantic Water transported northward by the Western Spitzbergen Current, divides into two currents. One of these currents continues northward and the other travels to the west creating turbulence^51^ and upwelling. Sperm whale presence has been shown to be correlated with increased circulation and turbulence^52,53^. These conditions typically promote upwelling and species richness and hence potential prey availability, which likely explains the highest occurrence in our study of sperm whales in Eastern Fram Strait. Similarly, the low yet consistent, multi-year presence at Atwain may be explained by both suitable bathymetry (shelf-edge) and the influence of the Western Spitsbergen Current, which keeps the sea ice cover relatively low. However, sea ice extent at Atwain varied significantly interannually^54^. During the data collection periods in this study, ice cover was generally low, but between 2014 and 2015 the area was covered in very close drift ice practically year-round, but unfortunately no acoustic data was collected from that period (Appendix Figure A4). Previously, single instances of acoustic sperm whale presence have been reported from Atwain^15^, but a consistent seasonal presence was not expected based on previous observational studies and habitat suitability models^17^. These contrasting results highlight the importance of combining both visual and acoustic methods^55^, especially for deep divers like sperm whales in areas that are difficult to access. The Isfjorden mooring, although on the shelf and in shallower water, is also exposed to an in-and-out circulation flow of Atlantic Water onto the shelf via the Isfjorden Trough, a trench deeper than 200 m, that creates a hybrid area between shelf and shelf edge waters^56,57^. Despite low ice cover and some Atlantic Water influence, sperm whale occurrence was scarce and inconsistent in Kongsfjorden. A recent study reported an increase in sightings near the mouth of Kongsfjorden, so more acoustic detections could have been expected^19^. The location of the hydrophone approx. 15 km into the fjord rather than at the mouth might reduce the reception of acoustic signals coming from outside of the fjord. Nonetheless, the present results are similar to Llobet et al.^58^ that found sperm whale vocal presence only on 1 day a year in this fjord. It is important to note, that the Eastern Fram Strait hydrophones were deployed significantly deeper (over 800 m) than the other sites (around 200 m) which might also contribute to the increased presence at Eastern Fram Strait, given that sperm whales are deep divers.

The timing of sperm whale migrations to the high latitude feeding grounds is not known with any degree of precision, but on-going tagging studies show quite asynchronous migration times for males (unpublished NPI/UiT data) with departures in the autumn or early winter being the norm. Sperm whale diet is believed to mainly consist of cephalopods and fish, but there is regional variation. Boreoatlantic armhook squid (*Gonatus fabricii)* has been identified as an important prey species in the North Sea^59^ and Northern Norway^60^ and this potential prey species does range up to northern Svalbard (Atwain). High concentrations have been reported around the Eastern Fram Strait site^61^ which is also a suspected spawning site for these squid^62^. MacKenzie et al.^63^ concluded based on their stable isotope analyses that sperm whales likely consume deep-water species such as squid. Fish species such as the lumpsucker (*Cyclopterus lumpus*) and Atlantic cod (*Gadus morhua*) have also been found to be part of sperm whale diet in the North Atlantic^60,64^ and these fish species are also found in the Eastern Fram Strait and off the west coast of Spitsbergen^65,66^. At Eastern Fram Strait, sperm whale presence peaked shortly after the peak in zooplankton concentration. This may indicate that sperm whale prey likely benefitted from the zooplankton blooms. Atlantic Water circulation via the Isfjorden Trough is strongest in the winter^56,57^, which might increase prey availability in the area at that time and explain the peak in sperm whale presence late in the year. Randelhoff et al.^67^ demonstrated that “potentially upwelling-favorable” winds north of Svalbard occur from October to April, which could explain sperm whale detections peaking late in the year at Atwain, while there were none in the summer or earlier in the autumn, despite practically ice-free conditions. Sperm whales in the Northern Hemisphere are known to breed between January and August^68^, which may explain their absence or very low presence in high latitude regions during spring and early summer.

In terms of diel patterns, in the day-night season at both Atwain and Eastern Fram Strait, most detections occurred during twilight (dusk or dawn) and daylight, and a low number of detections occurred during the night. It may be that sperm whales adjust their foraging behavior to diel movements of potential prey species. For example, the Boreoatlantic armhook squid performs diurnal vertical migrations^69^. It has been shown that sperm whales adjust their foraging strategy to optimize the relationship between prey energetic value and the foraging energy cost at different depths or prey layers^48^. It may be that the most energetically valuable prey is found in deeper waters and less scattered in the water column vertically during the day, allowing for more efficient foraging. During the Polar Night, there is a weak signal for increased occurrence of sperm whales from 18:00 to 01:00 at Atwain. However, more data would be needed to confirm these suggested patterns. Previous studies have reported contrasting diel patterns or an absence of diel patterns in sperm whale presence globally^35,37,70^.

Usual clicks, used for navigation and foraging, were the most common call type recorded. Despite a positive bias towards detection of usual clicks over other click types in this study, this result is expected given that the sperm whales are in these high latitude areas for foraging purposes. Buzz clicks, associated with foraging and prey capture attempts^71^, were detected at similar rates at Isfjorden and Eastern Fram Strait but at a lower rate than the other click types. However, the occurrence of buzz clicks is likely underestimated due to the limited detectability of these highly directional clicks with remote hydrophones^30^. Male sperm whales are considered to be increasingly solitary with age and it is older males that travel furthest north^5^. However, given that slow clicks are thought to serve a social, communication function^35,36^, the prevalence of slow clicks at the study locations even at the northernmost mooring (81° N) suggests that male sperm whales at high latitudes may be more social than previously thought. Indeed, the proximity of males during tagging efforts in the west coast shelf off Spitsbergen suggests that loose social groups do exist, with males clearly being within acoustic range of each other, and not spread randomly (K.M. Kovacs, C. Lydersen, personal observations). This is further supported by observations of overlapping click trains from more than one individual in at least half of the recordings. It is unknown whether food densities alone dictate these groupings, or whether males are social with one another. The relative occurrence of slow clicks in Eastern Fram Strait compared to Isfjorden and Atwain was low, which might indicate that Eastern Fram Strait is a feeding hotspot, and less time is allocated on non-foraging activities, like socializing or communication.

## Conclusion

This study provides a multi-year, year-round baseline for sperm whale acoustic presence around the Svalbard Archipelago. Sperm whale presence was detected at most recording sites with the greatest presence at deep, shelf edge areas with low sea ice cover. The highest occurrence rates were found off western Svalbard, where sperm whale vocal activity was detected almost year-round. Even the northernmost location at 81° N demonstrated a consistent, reoccurring acoustic presence. In addition, the occurrence of slow clicks and overlapping vocalizations from multiple individuals supports the idea of loose aggregations of sperm whales rather than solitary behavior in the High Arctic. While some seasonality of detections was demonstrated, future studies should focus more on the drivers of the timing of sperm whale presence. Prey availability might play a role in the timing of their winter departures, but mating or other explanations are also plausible. With the increasing loss of sea ice in the High Arctic, the already documented northward shift of the sperm whales’ geographical range in the Svalbard area is likely to continue as new areas become accessible. The present study provides a baseline for future PAM-monitoring of acoustic presence of sperm whales around Svalbard. Research based on long-term monitoring will be especially crucial to predict cetacean community changes and to advise management authorities given the rapidly changing environment.

## Methods

### Data collection and deployment locations

This study used underwater passive acoustic data collected at eight different locations around the Svalbard Archipelago (Fig. 1). At each location, an autonomous underwater acoustic recorder was installed on an oceanographic mooring. These moorings were deployed and maintained as part of several long-term monitoring programs^72–79^. Eastern Fram Strait (EFS) moorings were maintained by the Alfred Wegener Institute Helmholtz Centre for Polar and Marine Research (Bremerhaven, Germany); at these sites SonoVault recorders (SonoVault, develogic GmbH; RESON TC4037-3 hydrophone with a sensitivity of – 193 dB and a frequency response from 8 Hz to 48 kHz) were used. These moorings were located approximately 25 km apart, but for the data analysis they were considered as one location. At the rest of the moorings, AURALs (AURAL M2, Multi-Électronique Inc.; HTI-96-MIN hydrophone with receiving sensitivity of − 164 ± 1 dB re1 V μPa − 1 and frequency response from 2 Hz to 30 kHz) were used by the Norwegian Polar Institute (Tromsø, Norway). The Atwain mooring was moved after the first two recording years, in 2015, to shallower water, approximately 50 km away from the original location. The Isfjorden mooring was also moved after the first two years, to a site approximately 25 km away from the original location, deeper into the fjord. Despite small interannual differences in exact mooring locations and depths within recording sites, the years for each site were grouped for the analyses, given that this study aimed to investigate distribution of sperm whales first and foremost on a broad scale around Svalbard. The acoustic data reported in this study spans a 10-year period from 2012 to 2021; data was collected for at least two recording periods at each location. Sampling rate, duty cycle and recording period varied slightly between locations and years. The recorders at EFS recorded acoustic data continuously^80^. For this study, the data was subsampled by taking only the first 10 min of every hour to correspond with the duty cycle-based sampling schemes of the data from the other mooring sites. Due to a recorder software issue at Eastern Svalbard 1, about 70% of the data between February and July 2020 was lost at that location (Appendix Figure A2). Full deployment details and coordinates for each location and year are presented in Table 1.

**Table 1:** Mooring metadata: Data sources: 1 = Norwegian Polar Institute, Tromsø, Norway, 2 = the Ocean Acoustics Group of the Alfred Wegener Institute Helmholtz Centre for Polar and Marine Research (Bremerhaven, Germany).

Location	Recording start	Recording end	Latitude	Longitude	Deployment depth (m)/bottom depth (m)	Sample rate (kHz)	Duty cycle (min/h) = duration of one recording	Total number of recordings	Data source
Eastern Svalbard 1	17/11/2019	24/07/2020	79.582917	28.073	60/252	32	12	3471	1
Eastern Svalbard 1	25/02/2021	10/11/2021	79.5839	28.065617	60/252	32	12	6201	1
Eastern Svalbard 2	03/10/2018	29/06/2019	79.678833	32.319833	64/357	32	12	6462	1
Eastern Svalbard 2	17/11/2019	23/09/2020	79.6756	32.314733	67/350	32	12	7463	1
Eastern Svalbard 2	01/10/2020	11/11/2021	79.6756	32.314733	67/360	32	12	9757	1
Atwain	18/09/2012	04/05/2013	81.543167	30.856667	55/850	32	17	5463	1
Atwain	22/09/2013	02/05/2014	81.544583	30.85345	59/850	32	17	5327	1
Atwain	19/09/2015	05/12/2016	81.40425	31.22555	57/200	32	12	10,622	1
Atwain	25/09/2017	08/12/2018	81.40978	31.241767	55/206	32	12	10,522	1
Eastern Fram Strait	23/07/2016	18/07/2017	79.0002	5.6687	808/2083	48	10	8725	2
Eastern Fram Strait	20/08/2018	01/08/2019	79.1665	6.3327	836/1414	48	10	8425	2
Western Fram Strait	02/09/2012	11/04/2013	78.799533	− 4.9932	75/1014	32	17	5264	1
Western Fram Strait	08/09/2013	27/04/2014	78.833967	− 4.993183	76/1015	32	17	5532	1
Western Fram Strait	07/09/2015	30/08/2016	78.836067	− 5.001433	74/1010	32	12	8583	1
Western Fram Strait	09/09/2016	27/08/2017	78.836067	− 5.001433	84/1018	32	12	8433	1
Western Fram Strait	10/09/2017	28/08/2018	78.83805	− 4.986517	71/1022	32	12	8437	1
Western Fram Strait	07/09/2018	03/09/2019	78.838733	− 5.002433	81/1022	32	12	8668	1
Isfjorden	05/10/2017	24/08/2018	78.18185	13.389167	50/242	32	12	7760	1
Isfjorden	29/08/2018	18/05/2019	78.18185	13.389167	50/242	32	12	6303	1
Isfjorden	08/09/2019	08/06/2020	78.125283	14.419283	42/160	32	12	6586	1
Kongsfjorden	09/10/2013	24/03/2014	78.96245	11.805033	51/230	32	17	3968	1
Kongsfjorden	25/09/2014	15/08/2015	78.962667	11.79725	33/223	32	15	7759	1
Kongsfjorden	21/09/2015	02/04/2016	78.9589	11.823833	57/223	32	12	4680	1
Kongsfjorden	31/08/2016	08/08/2017	78.959167	11.823717	50/223	32	6	8251	1
Kongsfjorden	18/08/2017	17/08/2018	78.958933	11.823933	58/224	32	15	8745	1
Rijpfjorden	20/09/2015	26/08/2016	80.294525	22.30254	52/227	32	12	8208	1
Rijpfjorden	14/08/2018	17/02/2019	80.294433	22.299183	63/240	32	15	4494	1

Duty cycle refers to the duration of each single recording in minutes, recorded from the start of every hour.

### Detection of sperm whale acoustic presence

#### Click detection and classification

A combination of automated and manual methods was used to detect sperm whale presence in the acoustic recordings (Appendix Figure B1). First, an automated detector was used to identify recordings containing potential sperm whale vocalizations. Note, that recording durations vary across sites and/or deployment years, see Table 1 for duty cycles. The automated detector setup combined two tools—an open-source bioacoustics software called PAMGuard (Version 2.02.02; available at http://www.pamguard.org)^81^ in combination with the R statistical program package (Version 4.2.). The first part consisted of processing sound recordings in PAMGuard using frequency band filters and the click detector module including click classifiers. First, a high pass frequency band filter (12th order Butterworth at 1 kHz) was applied to reduce energy at low frequencies, which are typically outside the sperm whale clicks main frequency range. In addition, EFS recordings were filtered (low pass 12th order Butterworth at 16 kHz) to match the frequency range of the other locations’ recordings. Then, a trigger frequency filter for the click detector module was set at 2 kHz threshold (high pass 6th order Butterworth) because the main energy in sperm whale clicks is typically above this threshold. The PAMGuard Basic Click Detector parameters were kept at default values (Appendix Table B3), including the signal-to-noise ratio (10 dB) because they captured sperm whale clicks effectively during initial exploratory analyses. Clicks detected in the Basic Click Detector were then passed through a set of classifiers. PAMGuard click classification works in a hierarchical manner where classes are tested for a click detection starting from the top of the classifiers list until a match is found. Therefore, the first classifiers on the list were defined to capture and discard as many false detections (i.e., sounds from ice, other odontocetes, walruses, and mooring self-noise such as cable strumming) as possible by setting limits for frequency content (peak, width, energy control bands) and signal length. Then two additional classifiers were defined to capture potential sperm whale clicks, one for peak frequency above 4 kHz (“sperm whale high”) and the other one between 1.5 and 6 kHz for longer duration clicks (“sperm whale low”). The detector was not designed to distinguish between click types per se, but the division into two sperm whale classifiers helped to reduce false detections from other low frequency signals such as walrus knocks. For exact classifier settings, see Appendix Table B4. The output from PAMGuard (i.e., detections classified as potential sperm whale clicks) went through a final sorting step to further reduce false detections using a custom-made script in R. Sperm whale clicks are known to occur in sequences of a certain Inter-Click-Interval (ICI)^30^. Therefore, single clicks or tight click clusters (i.e., click sequences of very low ICI) were unlikely to originate from sperm whales. Consequently, in the last step, click detections were accepted only if they appeared in sufficient numbers within a time window. If 5–50 clicks of the first classifier class (“sperm whale high”) appeared within a 10 s window (corresponding to an ICI between 0.2 and 2 s) or between 3 and 15 detections within a 30 s window (corresponding to an ICI between 2 and 10 s) in the second class (“sperm whale low”), they were accepted as potential sperm whale clicks. The recordings in which these conditions were fulfilled were labeled with “presence”. Note that the detector aimed to identify the presence of sperm whale clicks in the recordings but not to quantify them.

In a post-processing step, all recordings labeled with “presence” were manually (i.e., visually and aurally) assessed by a trained human analyst to confirm sperm whale click presence using Adobe Audition software (Version 22.2.0.61**)**. Recordings that did not contain sperm whale clicks were removed from the results. Sperm whale presence was reported as occurrence rate (i.e. proportion of the total number of recordings per location and year that contained sperm whale vocalizations) as well as daily presence (given as number of hours per day), and hourly presence (indicated by 1 for acoustic presence or 0 for acoustic absence).The presence or absence of different click types was scored in each recording along with the presence (or absence) of overlapping click sequences produced by more than one individual. The presence of click types was reported as the proportion of recordings per each location to contain each click type.

#### Detector training and validation

The dataset used for detector training consisted of 40 recordings (of variable durations, see duty cycles in Table 1). After manually scanning through hundreds of recordings from the available data, 20 recordings that contained sperm whale clicks were selected and another 20 recordings that contained click-like sounds were chosen that would likely cause false positive detections. The recordings containing sperm whale clicks were additionally labelled with ‘strong’ or ‘faint’ to describe the quality of the clicks present. ‘Faint’ clicks had either a low signal-to-noise ratio, energy on a very narrow frequency range or they were too few (< 3 or < 5 per time-window depending on classifier class) to form a click train. These clicks likely resulted from the vocalizing individual being far away from the hydrophone or ‘off-axis’ (i.e., the animal not oriented towards the hydrophone) which can result in signal transmission loss and/or distortion. ‘Strong’ clicks contained energy on a broader frequency range and were more numerous, likely produced by individuals close to the hydrophone and/or heading to its direction (‘on-axis’). For examples of both categories, see Appendix Figures C7–C15. Systematic trials were performed with the training and test set where the click classifier parameters and the properties of the final sorting step in R were varied for each trial. The detector performance of sperm whale click presence was rated on the ability to label recordings correctly with 1 or 0, presence or absence of sperm whale clicks, respectively. Recall (= the proportion of recordings containing sperm whale clicks successfully detected) and precision (= the proportion of the recordings labelled “presence” actually containing sperm whale vocalizations) were used to decide on the final parameters for detector classifiers (Appendix Figure B2).

A randomly sampled ‘test set’ (1913 recordings or 1% of the data from each site) was used to further evaluate the detector performance, especially to assess the false positive rate (the proportion of recordings without sperm whale clicks labelled as “presence”) which reflected the manual inspection workload ahead. Due to the rare occurrence of sperm whales in this ‘test set’ (32 of 1913 recordings), an additional ‘validation set’ with a higher proportion of recordings containing sperm whale clicks was evaluated for recall. Recordings from two full months with some known sperm whale presence (October 2013 from Atwain and September 2016 from EFS, with a total of 623 of 1464 recordings containing sperm whale presence) were manually inspected and compared to detector results.

### Environmental covariates and statistical analyses

Environmental data were retrieved from reanalysis datasets using EU Copernicus Marine Service Information: chlorophyll mass concentration and net primary production of biomass were obtained in 0.25° × 0.25° spatial resolution^82^; zooplankton mass content expressed as carbon in sea water were obtained in 0.083° × 0.083° spatial resolution^83^; and sea surface temperature was obtained in 0.05° × 0.05° spatial resolution^84^. All variables were retrieved as daily means for all locations and recording years in R (packages *ncdf4*^85^ and *raster*^86^). Note that chlorophyll and net primary production data was not available from May 2020 onwards.

Previous studies report varying detection ranges for sperm whale clicks, with most estimates being around 50 km^30,37,38^. Based on this general estimate, all daily environmental data were averaged over a 50 km radius around each hydrophone mooring site using the coordinates for each deployment period. Daily sea ice data was retrieved from the Norwegian Meteorological Institute^87^ for each recording site. Daily ice cover for each location was computed using the same principles as in Llobet et al.^88^. Around each mooring location, 1000 random points were created within a 50 km radius. Each point was then associated with an ice cover category (< 10%, 10–40%, 40–70%, 70–90%, 90–100%, 100%) for each day and the proportion of points in each category was calculated. For the statistical models, a single variable was used to describe the sea ice cover, calculated as the proportion of the 50 km area that is covered with sea ice (any category > 10%). Sea floor elevation data came from the International Bathymetric Chart of the Arctic Ocean^89^ in 200 mm × 200 mm grid cell spacing. Sea floor depth, slope and terrain heterogeneity (Terrain Ruggedness Index) were computed for each mooring location and averaged over a 50 km radius area in R (package *raster*^86^)*.* The daily acoustic presence data was examined using a generalized linear model with a binomial distribution and logit-link function (package *glmmTMB* in R) to evaluate potential effects of daily concentrations of chlorophyll a, zooplankton, net primary production, sea surface temperature and sea ice extent on daily sperm whale presence. To correct for temporal autocorrelation, an ar1-correlation structure was included in the models. Additionally, the sea ice coverage was compared between locations and times with sperm whale acoustic presence and absence using a non-parametric Wilcoxon test. All environmental covariates and ice cover were plotted against sperm whale presence for visual examination for each location.

### Light regime and diel variations

Potential diel patterns in acoustic presence were also explored visually. Because of the very high latitude location of the recording sites, where sun exposure varies seasonally, detections were first divided into light regime categories by date: polar day (length of the day = 24 h, solar elevation is higher than 0° over the 24-h day), polar night (length of the day 0 h, solar elevation is lower than 0° over the 24-h day) and day-night season (length of day > 0 and < 24 h). The hourly distribution of acoustic presence in these three diel categories were compared. Then, in the day-night season category, every detection was associated with a sun exposure category: day (solar elevation higher than 0° at the start of the hour), twilight (solar elevation between 0° and − 24°) or night (solar elevation below − 24°).

### Supplementary Information

Supplementary Information 1.

Supplementary Information 2.

## Data Availability

Acoustic spectrograms depicting sperm whale vocalizations can be found in the Supplementary Material, accompanied by recordings (.wav-files) for each spectrogram. The sperm whale presence data and environmental data used in analyses and figures are deposited in the Norwegian Polar Data Centre: doi: 10.21334/npolar.2023.1cc4e28b. The acoustic data from the Eastern Fram Strait recorder (2016–2017 deployment period) is available at doi:10.1594/PANGAEA.945404 (Thomisch et al.^80^) via the data repository PANGAEA.
