# Supplementary material for: High Arctic “hotspots” for sperm whales (Physeter macrocephalus) off western and northern Svalbard, Norway, revealed by multi-year Passive Acoustic Monitoring (PAM)
Source: Sci Rep. 2024 Mar 9;14:5825. doi: 10.1038/s41598-024-56287-9 (PMC10924940; doi:10.1038/s41598-024-56287-9)
Supplement: Supplementary file 2 — Supplementary Information 2. [file 41598_2024_56287_MOESM2_ESM.pdf]

High Arctic "hotspots" for sperm whales (*Physeter macrocephalus*) off western and northern Svalbard, Norway, revealed by multi-year Passive Acoustic Monitoring (PAM)

Viivi Pöyhönen[1], Karolin Thomisch[2], Kit M. Kovacs[1], Christian Lydersen[1], Heidi Ahonen[1]

A. Detected sperm whale presence.

Table A 1: Summary of sperm whale detections across locations and deployment years.

Location	Recording year (in the 2000s)	Number of recordings with sperm whale presence	Percentage of total recordings with sperm whale presence
Eastern Svalbard 1	19-20	0	0.00
Eastern Svalbard 1	20-21	0	0.00
Eastern Svalbard 2	18-19	0	0.00
Eastern Svalbard 2	19-20	0	0.00
Eastern Svalbard 2	20-21	6	0.06
Atwain	12-13	28	0.49
Atwain	13-14	54	0.98
Atwain	15-16	20	0.19
Atwain	17-18	40	0.38
Eastern Fram Strait	16-17	1450	16.62
Eastern Fram Strait	18-19	675	8.01
Western Fram Strait	16-17	0	0.00
Western Fram Strait	12-13	0	0.00
Western Fram Strait	13-14	0	0.00
Western Fram Strait	15-16	0	0.00
Western Fram Strait	17-18	0	0.00
Western Fram Strait	18-19	0	0.00
Isfjorden	17-18	144	1.86
Isfjorden	18-19	222	3.52
Isfjorden	19-20	105	1.59
Kongsfjorden	17-18	1	0.01
Kongsfjorden	16-17	0	0.00
Kongsfjorden	14-15	0	0.00
Kongsfjorden	13-14	7	0.18
Kongsfjorden	15-16	0	0.00
Rijpfjorden	15-16	11	0.13
Rijpfjorden	18-19	0	0.00

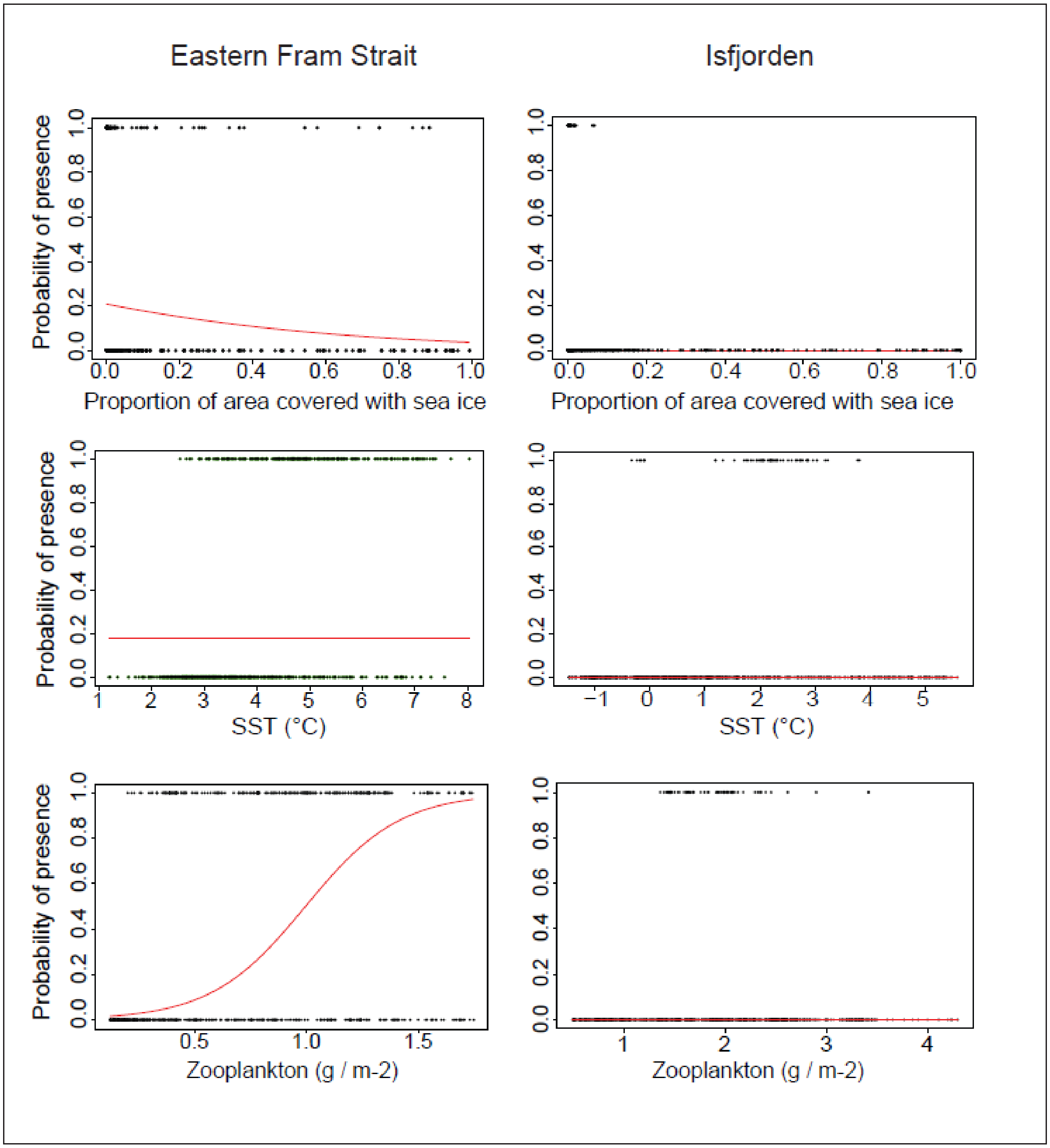

Figure A 1: Relationship between sperm whale presence and environmental variables within a 50km radius around the Eastern Fram Strait and Isfjorden recording sites based on logistic regression. Red line represents the predicted probability for sperm whale presence, and black dots illustrate the actual detected acoustic presence-absence of sperm whales. Note, that while the applied temporal autocorrelation structure (ar1) improved the model fit, some autocorrelation remained in the data.

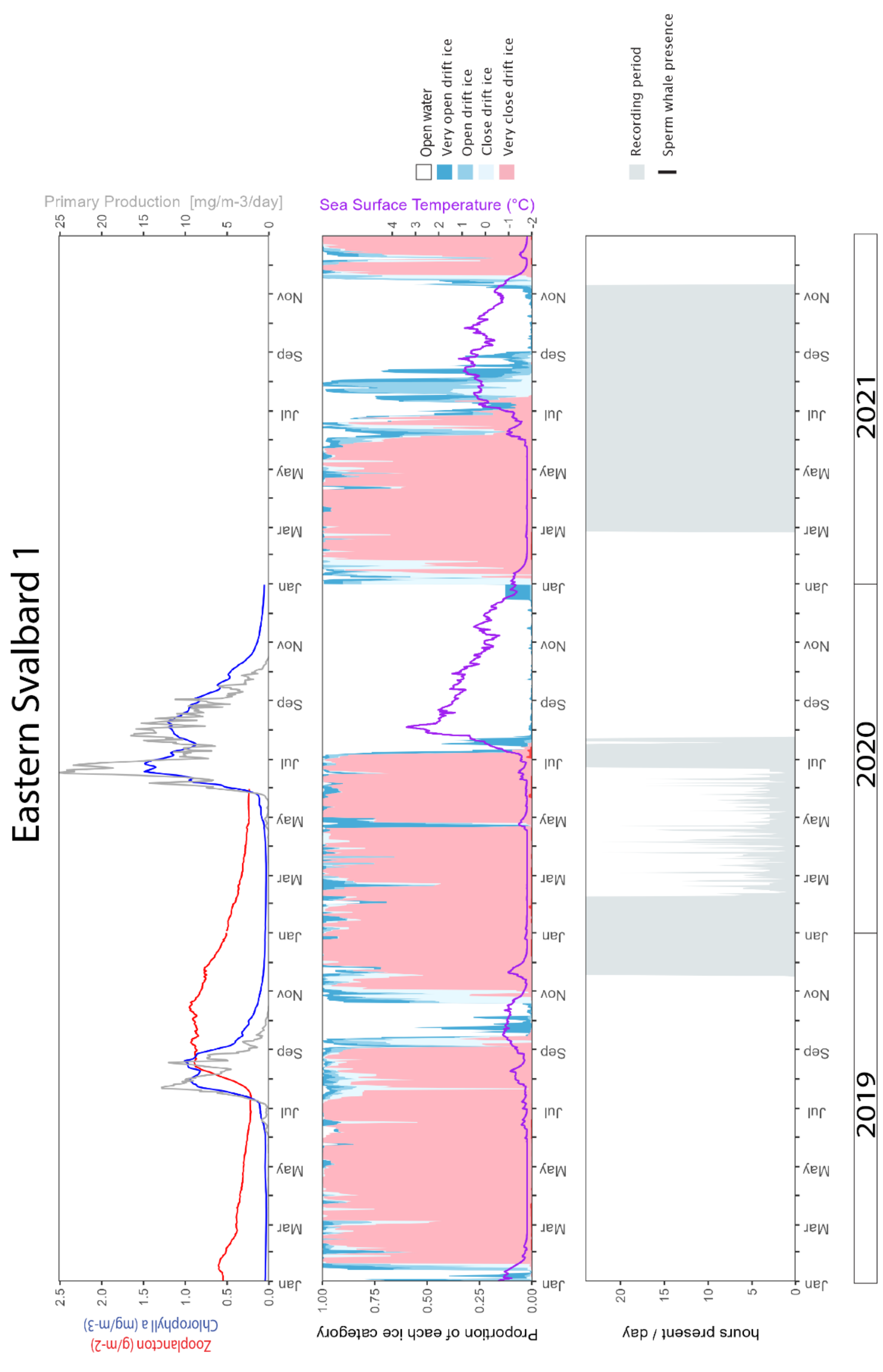

Figure A 2: Daily mean net primary production, zooplankton and chlorophyll a concentration, daily sea ice cover, daily mean sea surface temperature and sperm whale acoustic presence at Eastern Svalbard 1.

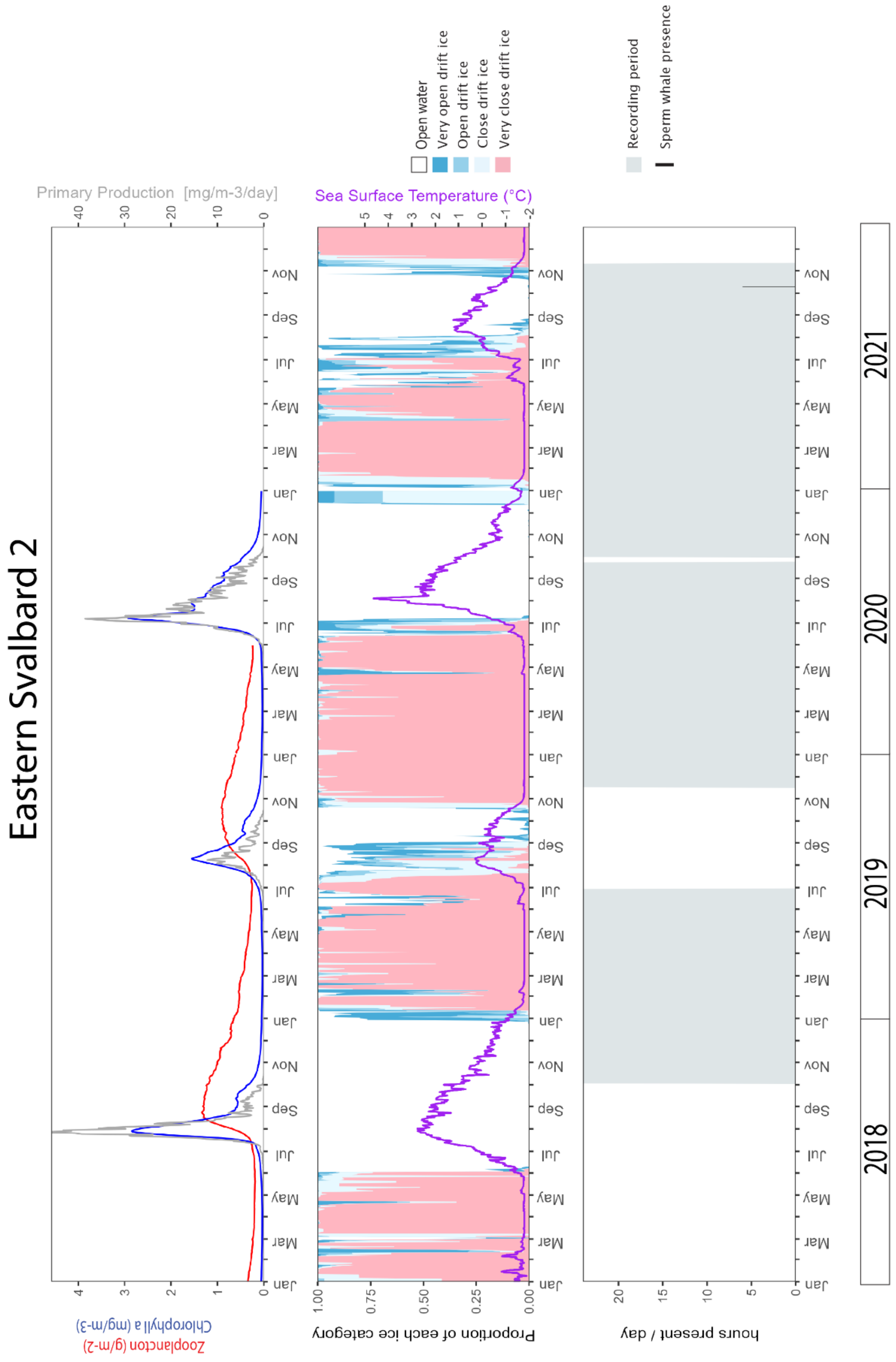

Figure A 3: Daily mean net primary production, zooplankton and chlorophyll a concentration, daily sea ice cover, daily mean sea surface temperature and sperm whale acoustic presence at Eastern Svalbard 2.

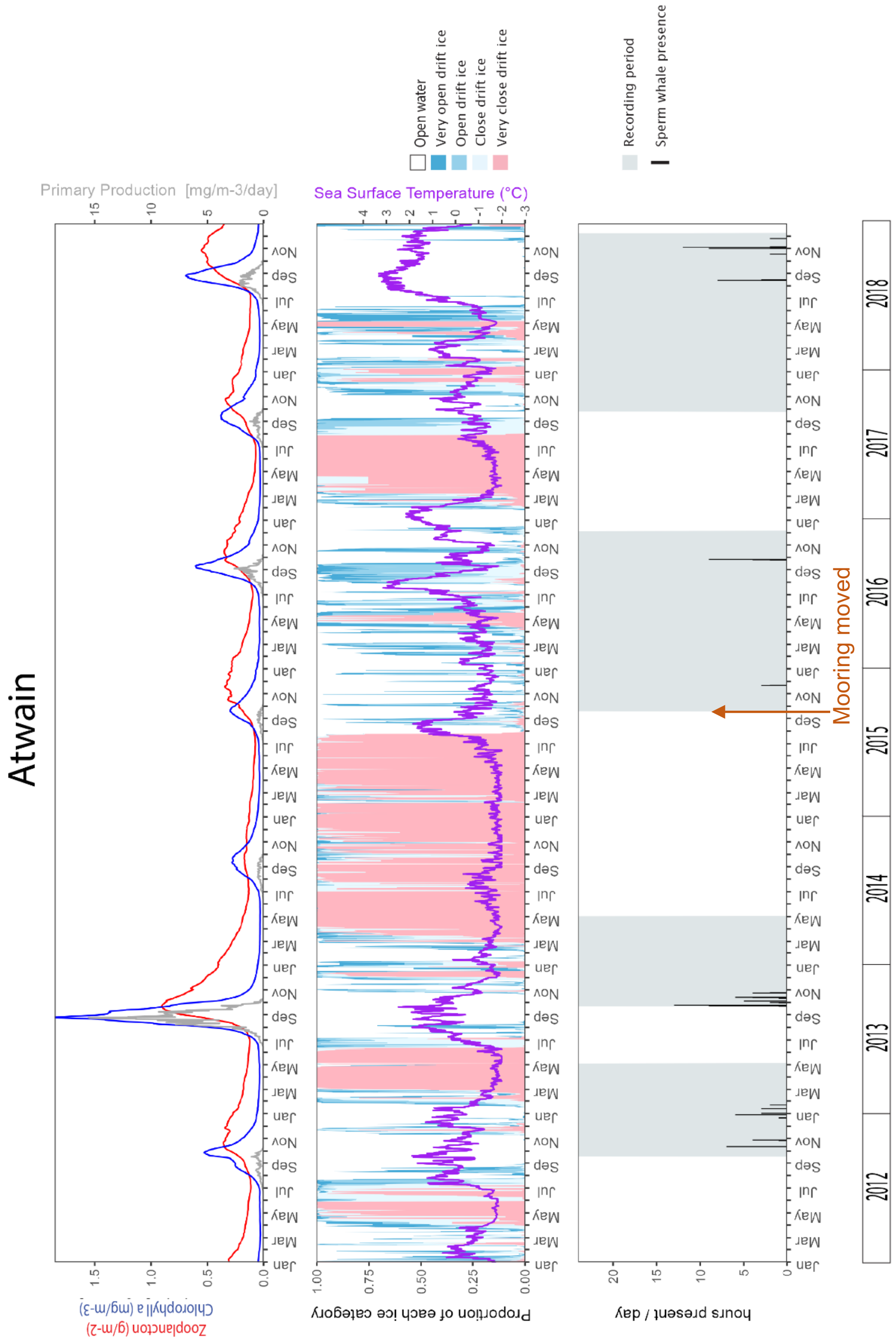

Figure A 4: Daily mean net primary production, zooplankton and chlorophyll a concentration, daily sea ice cover, daily mean sea surface temperature and sperm whale acoustic presence at Atwain.

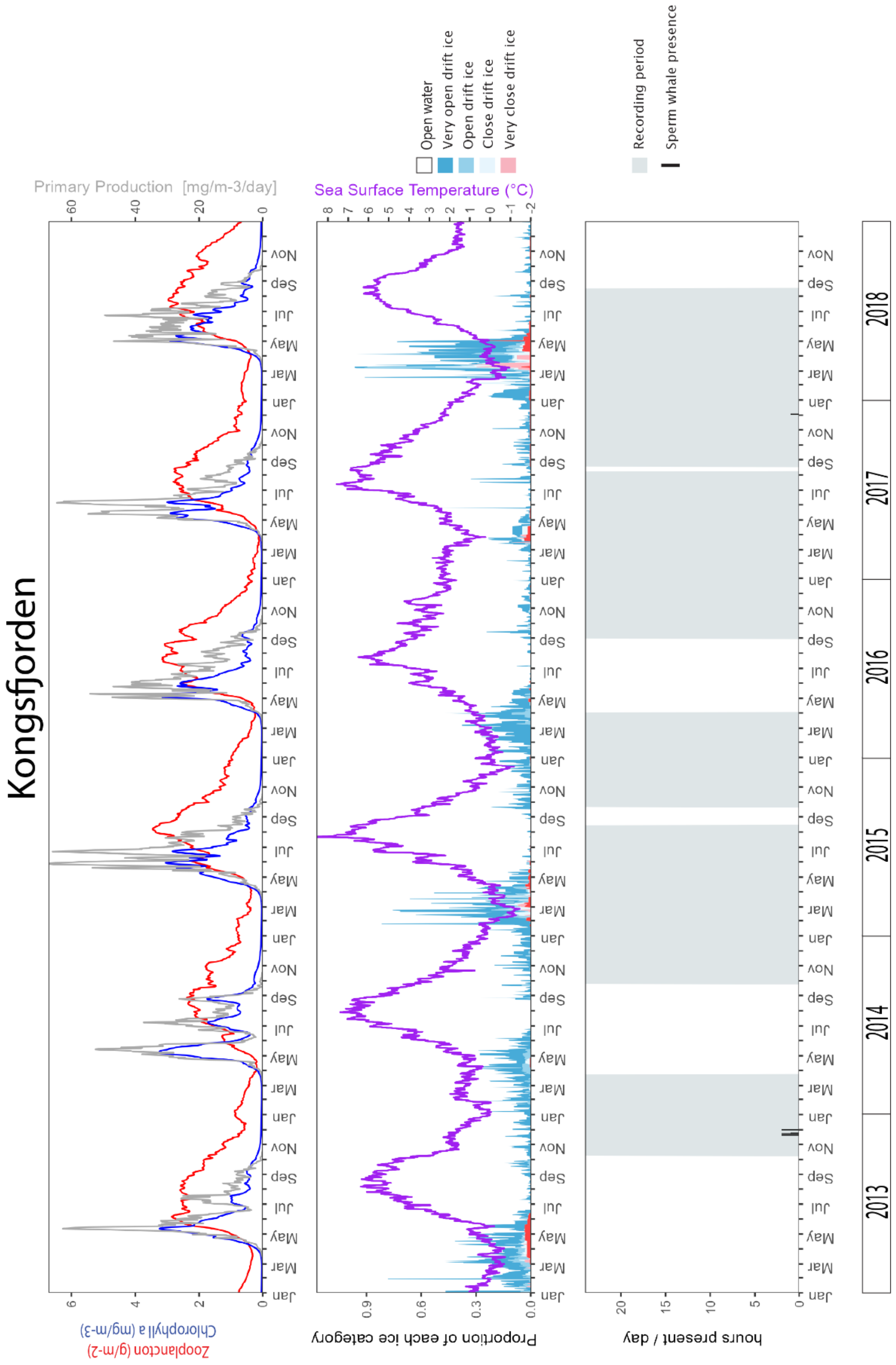

Figure A 5: Daily mean net primary production, zooplankton and chlorophyll a concentration, daily sea ice cover, daily mean sea surface temperature and sperm whale acoustic presence at Kongsfjorden.

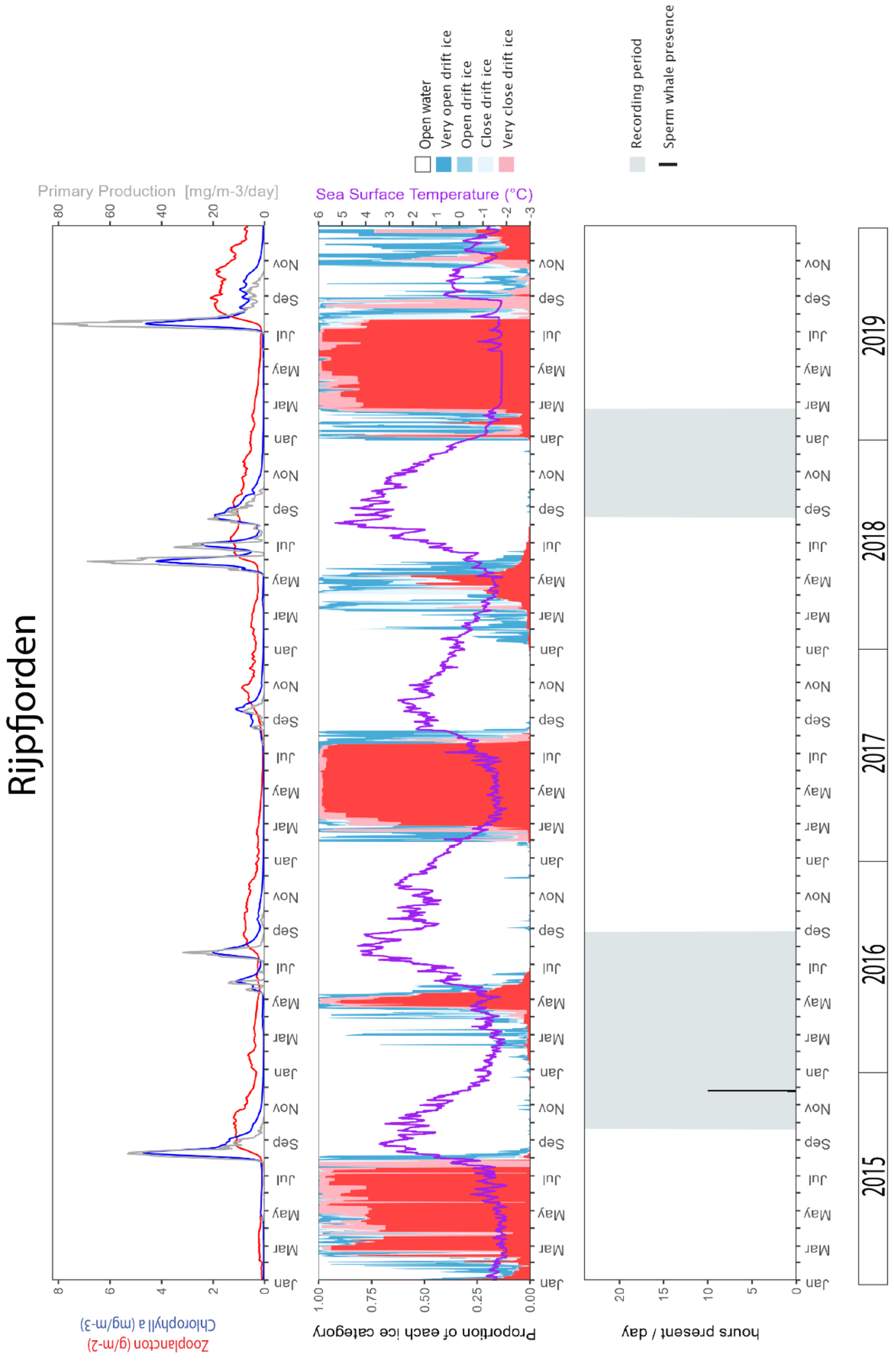

Figure A 6: Daily mean net primary production, zooplankton and chlorophyll a concentration, daily sea ice cover, daily mean sea surface temperature and sperm whale acoustic presence at Rijpfjorden.

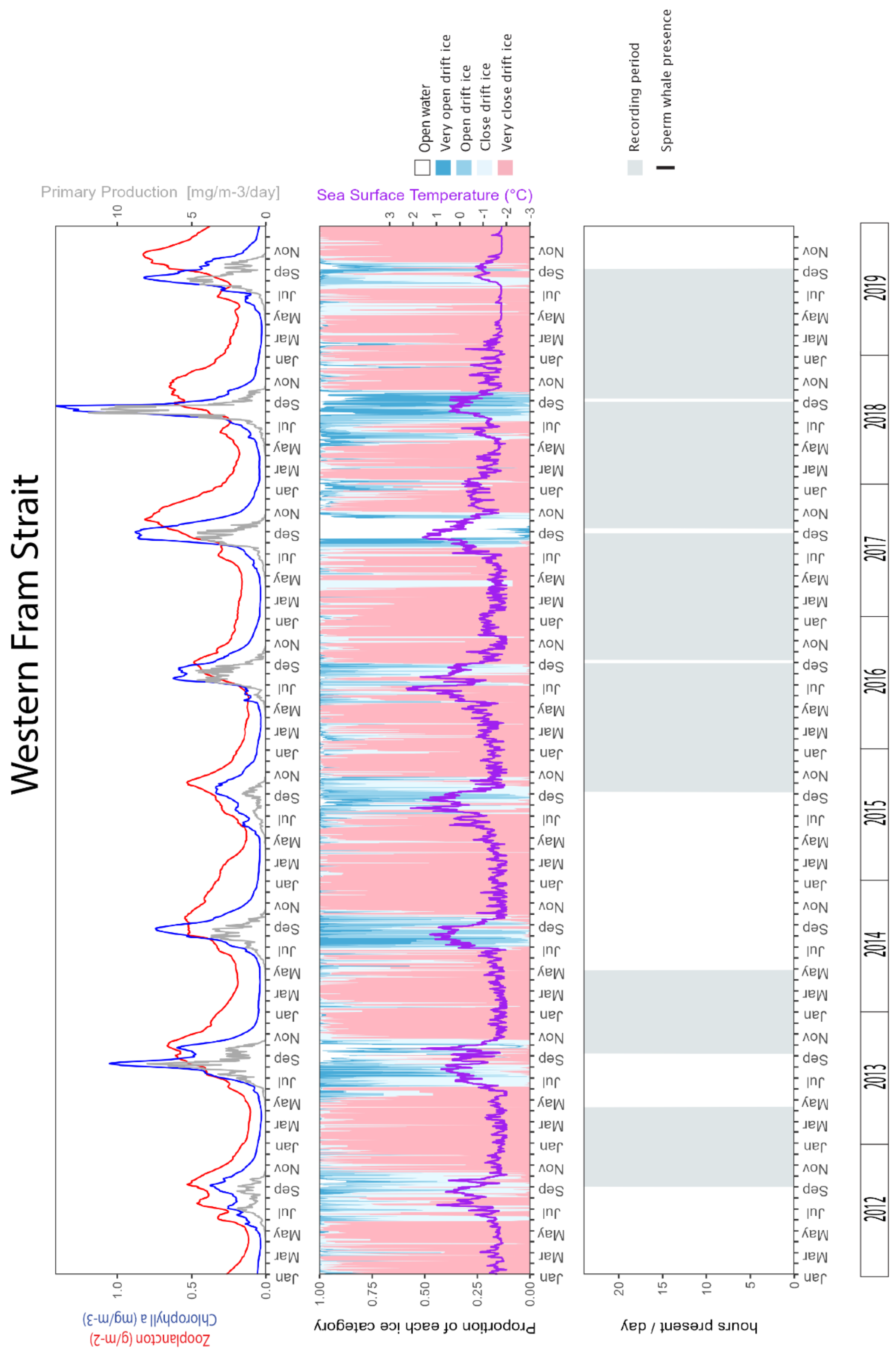

Figure A 7: Daily mean net primary production, zooplankton and chlorophyll a concentration, daily sea ice cover, daily mean sea surface temperature and sperm whale acoustic presence at Western Fram Strait.

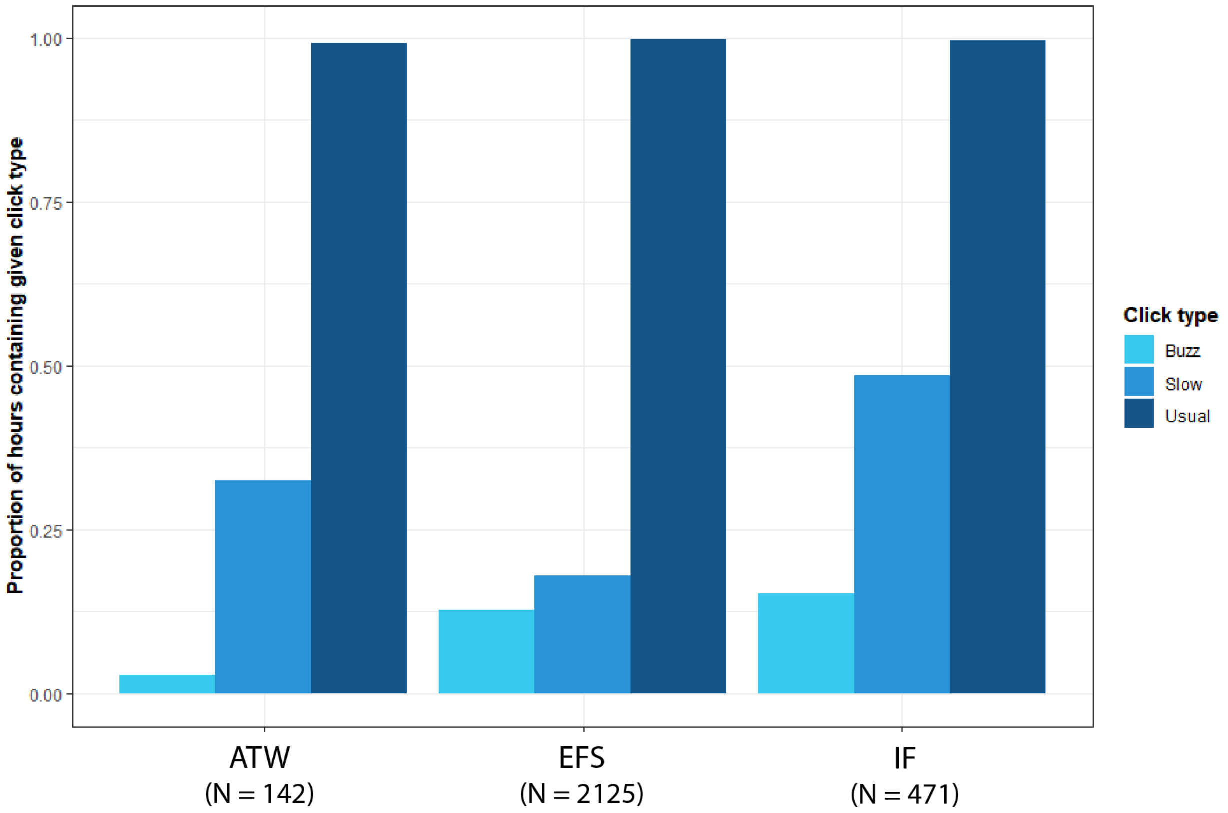

Figure A 8: Proportion of recordings containing each click type per location (all recording periods pooled). N = Total number of recordings containing sperm whale vocalizations. ATW = Atwain, EFS = Eastern Fram Strait, IF = Isfjorden.

B. Detector details

Table B 1: Automated detector recall (= percentage of recordings with sperm whale vocal presence successfully detected) in different datasets used during detector development.

	**Recordings containing vocalizations**		**Only recordings containing 'strong' vocalizations**	
Dataset	Number of recordings with sperm whale presence/total number of recordings	Recall (%)	Number of recordings with sperm whale presence/total number of recordings	Recall (%)
Training set	20/40	**75**	17/40	**92**
Test set (randomly sampled)	32/1931	**69**	19/1931	**89**
Validation set	623/1464	**64**	409/1464	**98**

Table B 2: Automated detector false positive rates (=percentage of recordings without sperm whale vocal presence incorrectly labelled with 'presence') evaluated with the 'test set' for each location.

**Location**	**Total number of recordings in 'test set'**	**False positive rate (%)**
ESV-1	192	20
ESV-2	472	11
ATW	638	15
EFS	328	0
IF	412	6
KF	668	0
RF	127	<1
WFS	724	50

Table B 3: PAMGuard Click Detector settings (default):

**Parameter**	**Values**
Threshold (SNR)	10 dB
Long filter	0.00001000
Long filter 2	0.00000100
Short filter	0.10000000
Minimum click separation (samples)	100
Maximum click separation (samples)	1024
Pre-sample	40

Post samples	40

Table B 4: PAMGuard Click Detector Click Classifiers parameters.

CLASSIFIER		Energy bands					Peak Frequency							Mean Freq				Click length		
		Test band (Hz)		Control band (Hz)		Min Energy Difference between test and control bands (dB)*	Search range (Hz)		Peak Range (Hz)		measure width over total energy (%)	Peak width range (Hz)		Summation range (Hz)		Selection range (Hz)		Measure width over total energy (%)	Click length range (ms)	
name	position	From	To	From	To		From	To	From	To		From	To	From	To	From	To		From	To
short_signals	1	NA	NA	NA	NA	NA	0	16000	0	16000	NA	NA	NA	NA	NA	NA	NA	99	0	4
narrowband	2	NA	NA	NA	NA	NA	0	16000	0	16000	50	0	1000	NA	NA	NA	NA	NA	NA	NA
low_band	3	0	5000	6000	7000	20	0	16000	0	5000	NA	NA	NA	NA	NA	NA	NA	NA	NA	NA
mid_band	4	10000	16000	0	10000	10	0	16000	10000	16000	NA	NA	NA	NA	NA	NA	NA	NA	NA	NA
walrus	5	0	3000	3000	16000	5	0	16000	500	3000	50	0	2000	0	16000	1000	3000	100	10	40
sperm_whale_high	7	6000	16000	0	3000	5	2000	16000	4000	16000	NA	NA	NA	1500	16000	4000	16000	NA	NA	NA
sperm_whale_low	8	1500	4000	10000	16000	10	1500	16000	1500	6000	NA	NA	NA	NA	NA	NA	NA	100	10	40

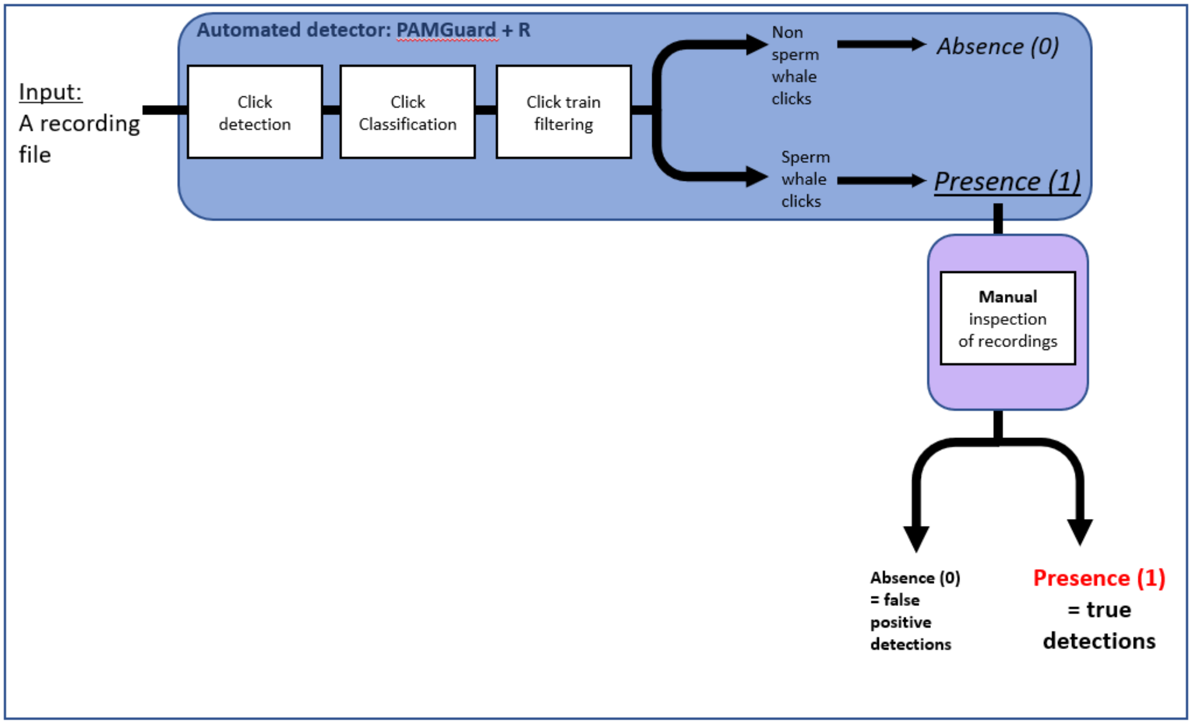

Figure B 1: An overview of the sperm whale presence detection process.

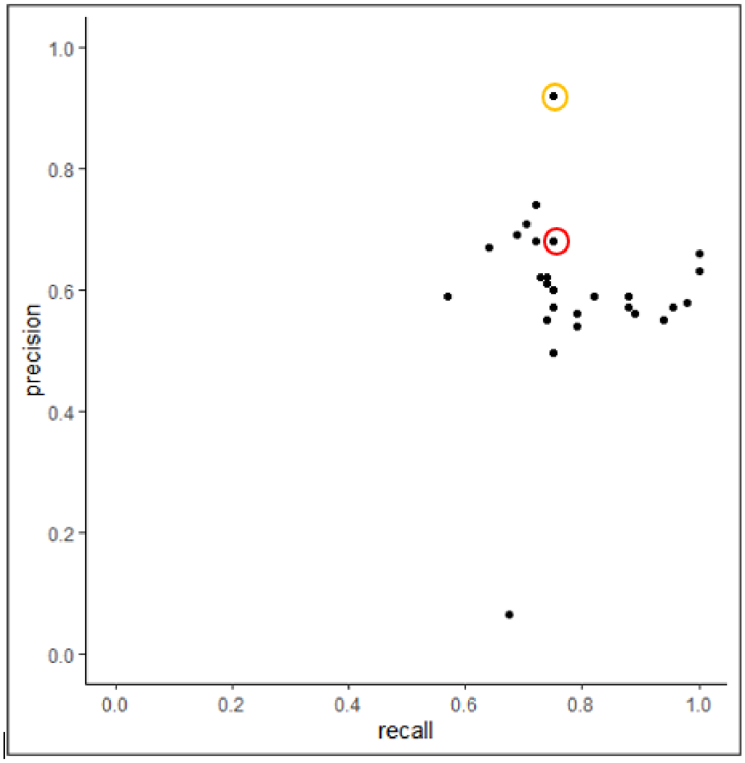

Figure B 2: Precision-Recall plot of trials with best performance on the 'training set'. Circled with red: performance with final detector parameters. Circled with orange: performance with final detector parameters when files containing only "faint" sperm whale clicks were removed.

C. Spectrograms

1. **Examples of likely false alarm triggers:**

<u>Ice-associated sounds</u>

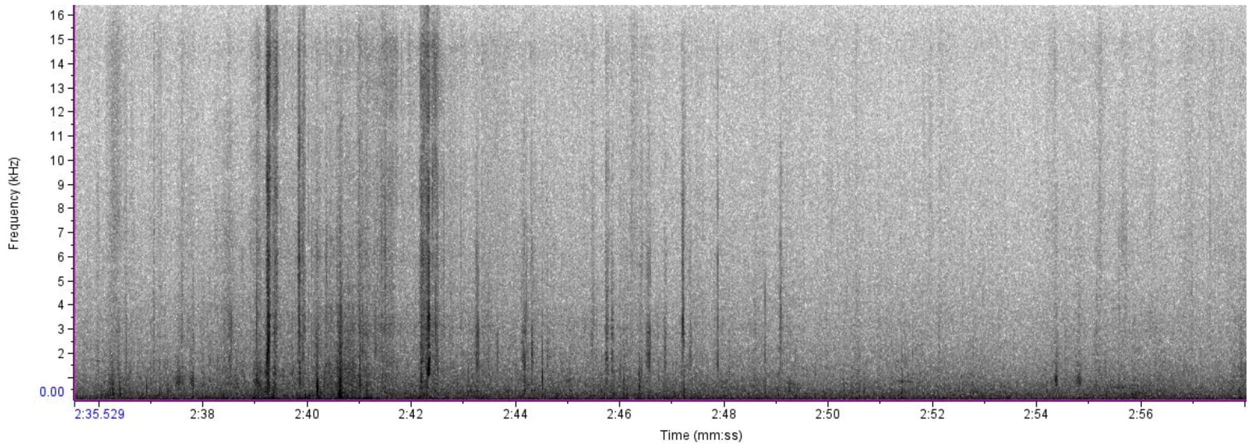

Figure C 1: Spectrogram showing click-like signals produced by ice (1024 FFT size, 50% overlap, Hann window).

<u>Self-noise</u>

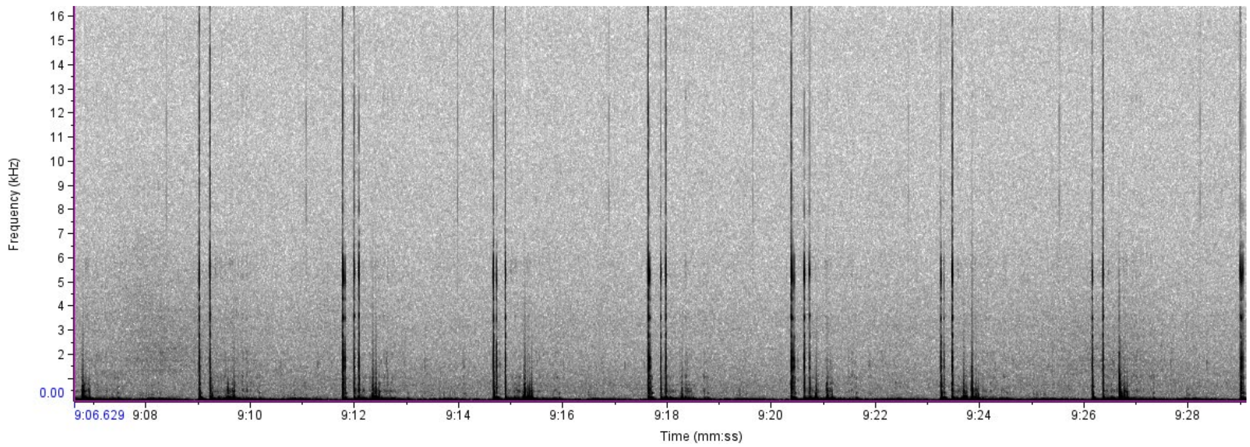

Figure C 2: Spectrogram showing self-noise from the instrument: likely cable-strumming on the hydrophone (1024 FFT size, 50% overlap, Hann window).

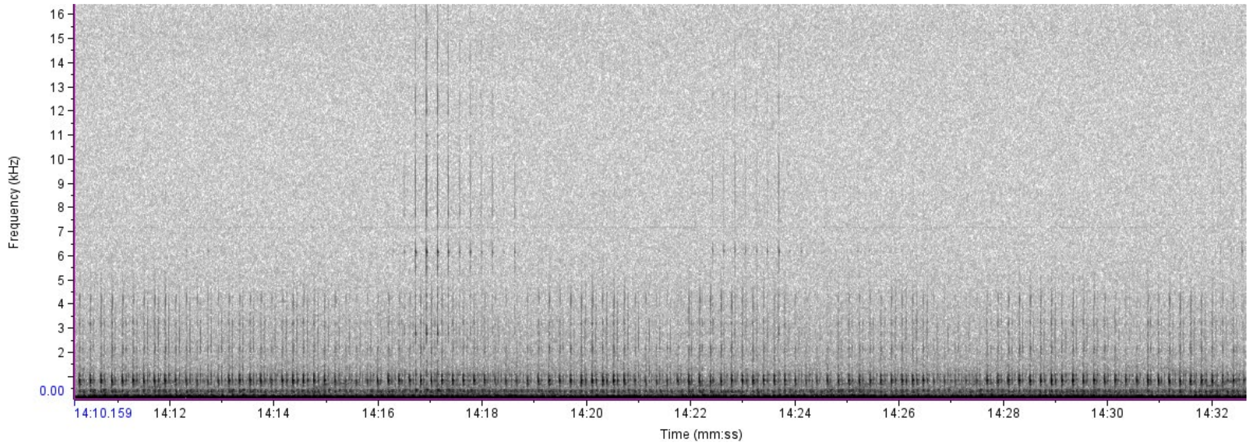

Figure C 3: Spectrogram showing self-noise from the instrument: likely cable-strumming on the hydrophone (1024 FFT size, 50% overlap, Hann window).

Walrus

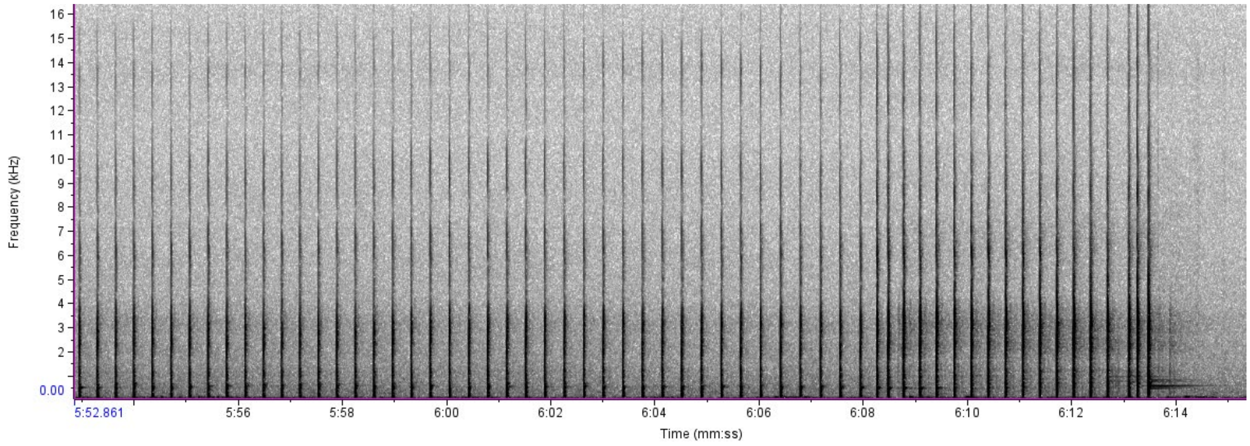

Figure C 4: Spectrogram showing walrus vocalizations (1024 FFT size, 50% overlap, Hann window).

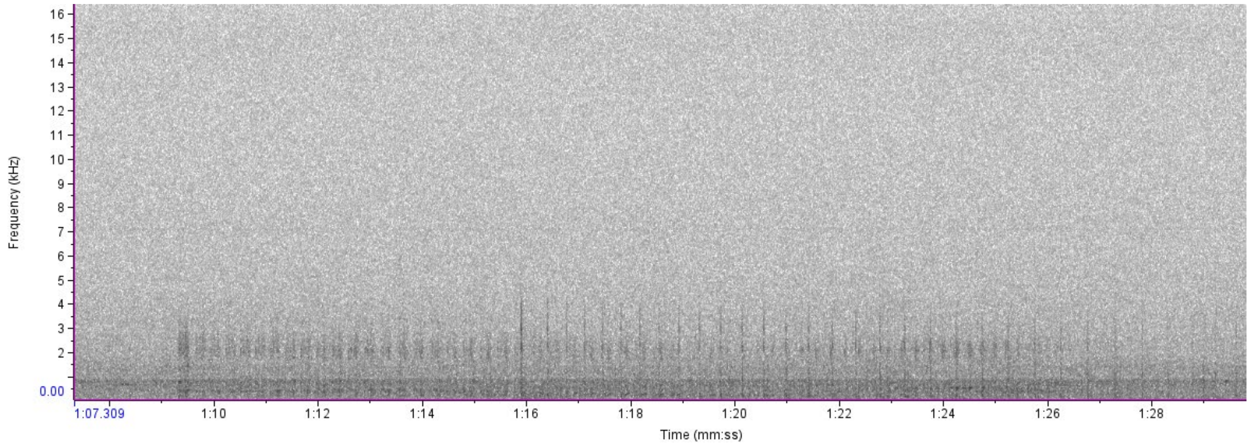

Figure C 5: Spectrogram showing walrus vocalizations (1024 FFT size, 50% overlap, Hann window).

Narwal

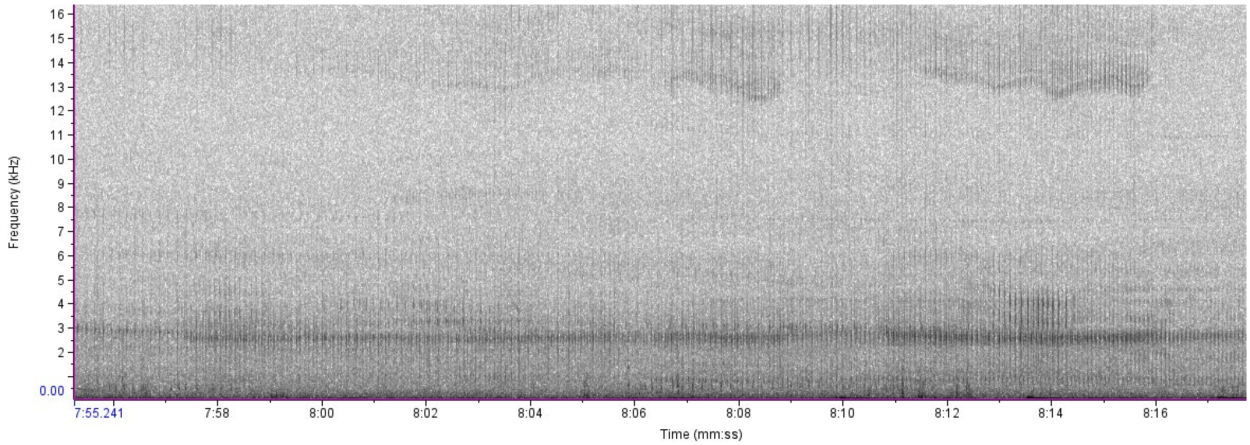

Figure C 6: Spectrogram showing narwal clicks (1024 FFT size, 50% overlap, Hann window).

Examples of detected sperm whale clicks:

“Strong” usual clicks

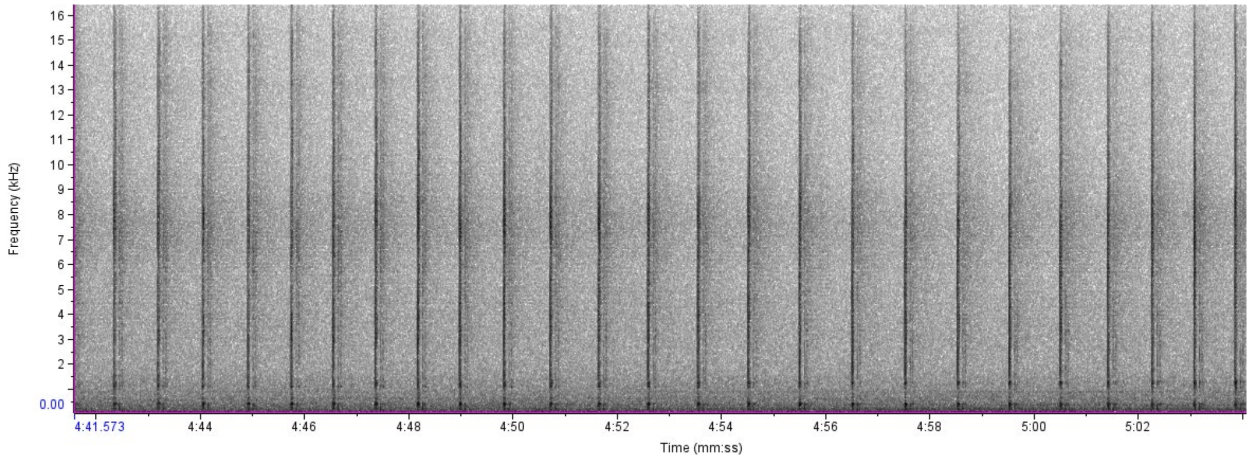

Figure C 7: Spectrogram showing ‘strong’ usual clicks (1024 FFT size, 50% overlap, Hann window) detected by the automated detector. For corresponding audio file, see “usual_clicks_in_suppl_FigureC7.wav” in supplementary files.

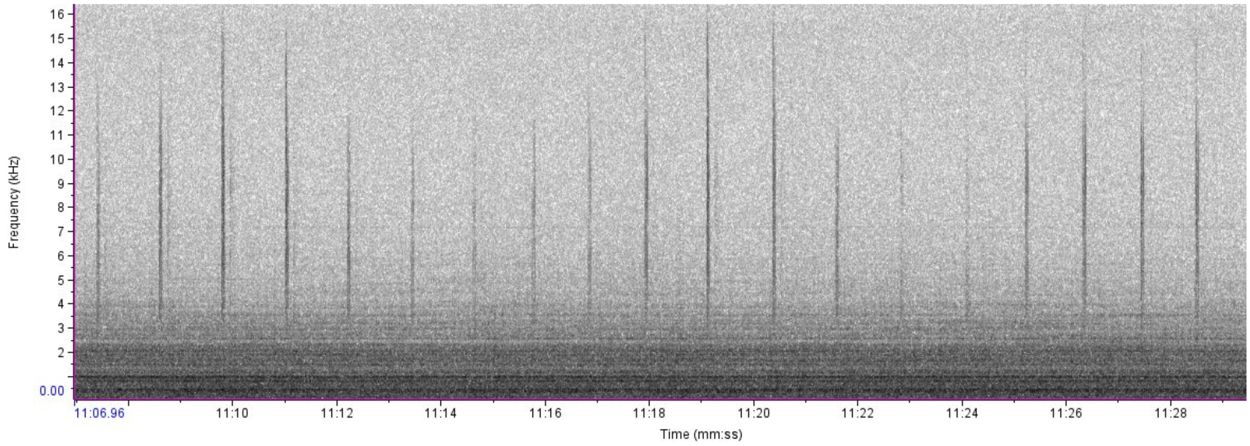

Figure C 8: Spectrogram showing 'strong' usual clicks (1024 FFT size, 50% overlap, Hann window) detected by the automated detector. For corresponding audio file, see "usual_clicks_in_suppl_FigureC8.wav" in supplementary files.

“Faint” usual clicks

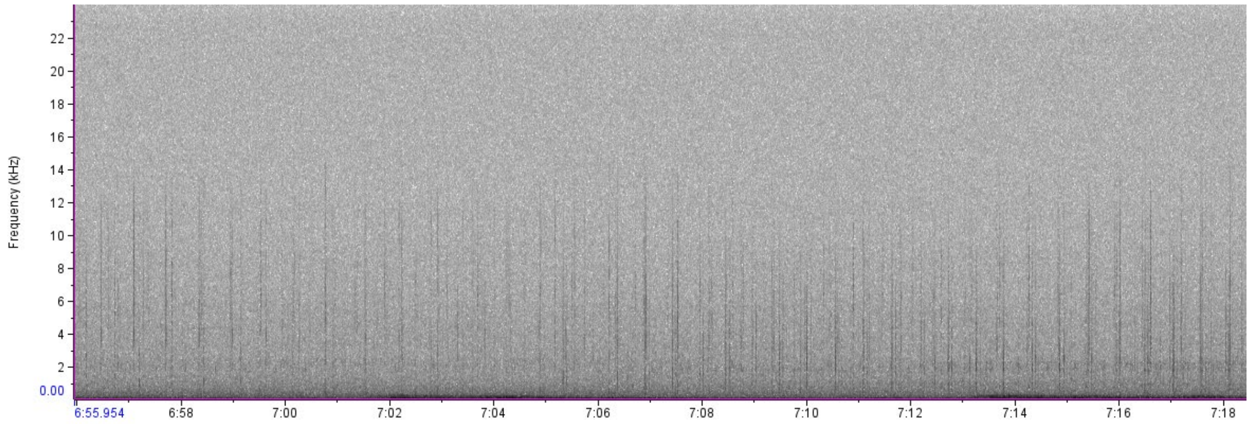

Figure C 9: Spectrogram showing ‘faint’ usual clicks (1024 FFT size, 50% overlap, Hann window) detected by the automated detector.

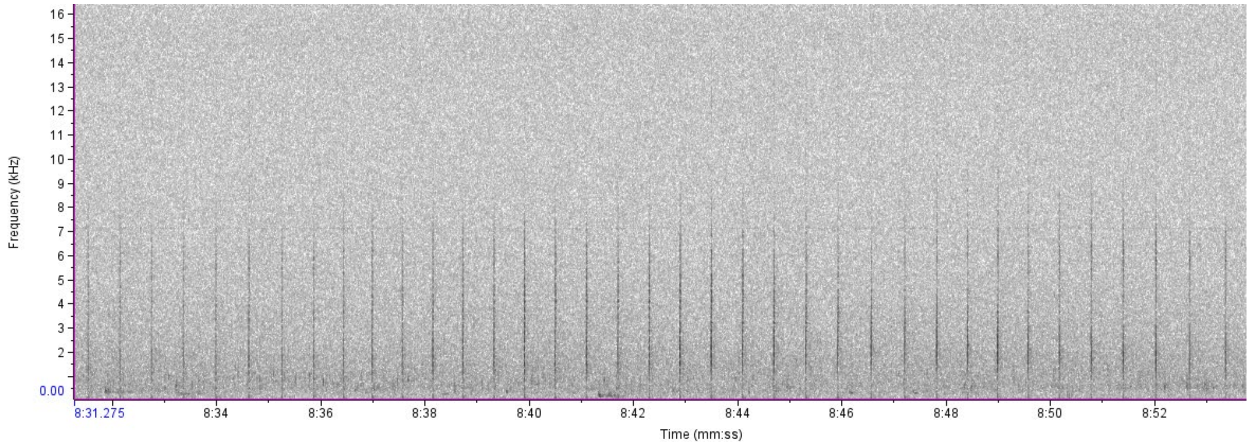

Figure C 10: Spectrogram showing ‘faint’ usual clicks (1024 FFT size, 50% overlap, Hann window) detected by the automated detector.

Slow clicks

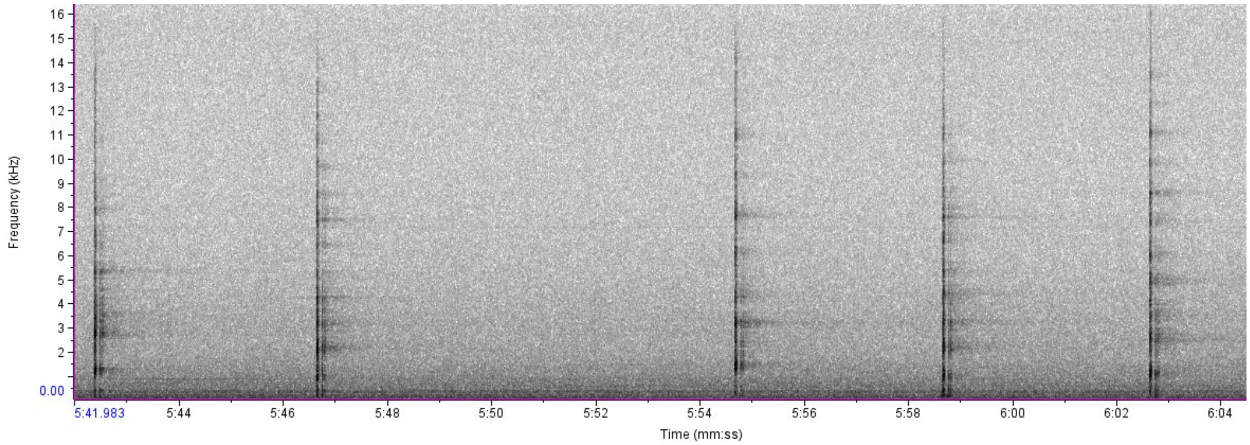

Figure C 11: Spectrogram showing slow clicks (1024 FFT size, 50% overlap, Hann window) detected by the automated detector. For corresponding audio file, see "slow_clicks_in_suppl_FigureC11.wav" in supplementary files.

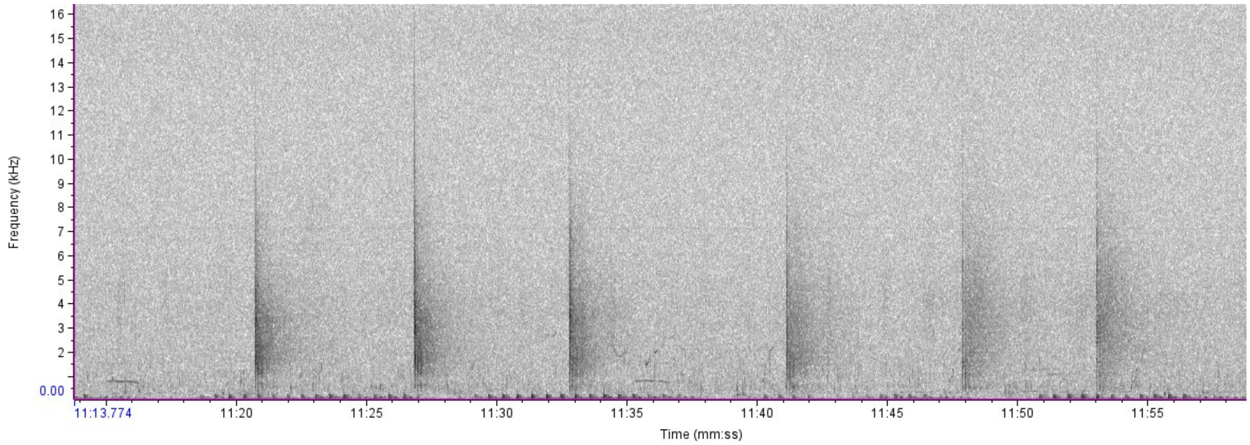

Figure C 12: Spectrogram showing slow clicks (1024 FFT size, 50% overlap, Hann window) detected by the automated detector. For corresponding audio file, see "slow_clicks_in_suppl_FigureC12.wav" in supplementary files.

Buzz clicks

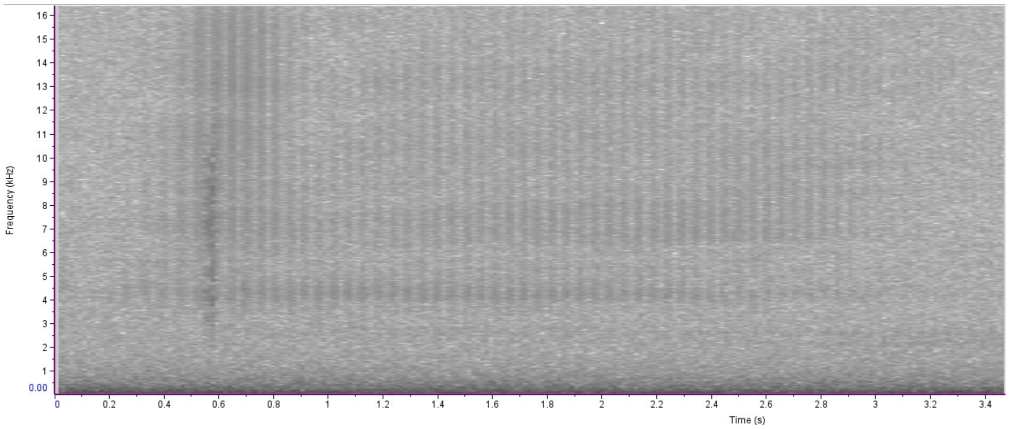

Figure C 13: Spectrogram showing buzz clicks (1024 FFT size, 50% overlap, Hann window). For corresponding audio file, see "buzz_clicks_in_suppl_FigureC13.wav" in supplementary files.

Overlapping clicks from more than one individual

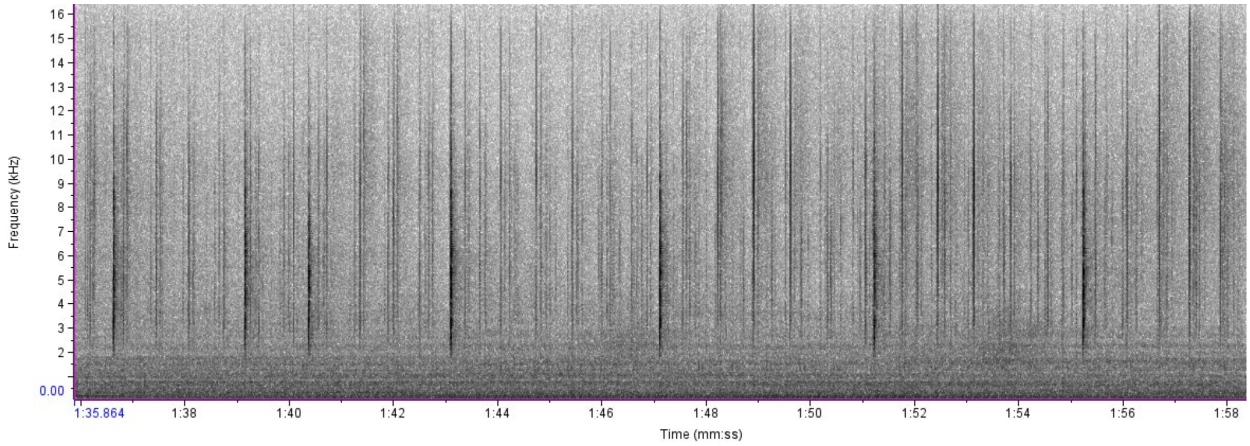

Figure C 14: Spectrogram showing overlapping clicks coming from more than one individual (1024 FFT size, 50% overlap, Hann window) detected by the automated detector.

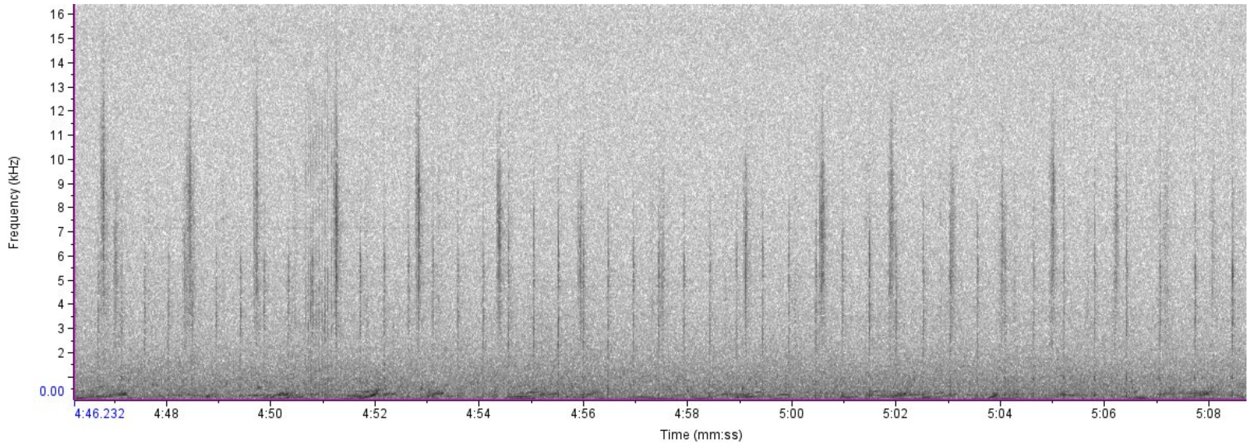

Figure C 15: Spectrogram showing overlapping clicks coming from more than one individual (1024 FFT size, 50% overlap, Hann window) detected by the automated detector.

2. Examples of missed sperm whale clicks (false negatives)

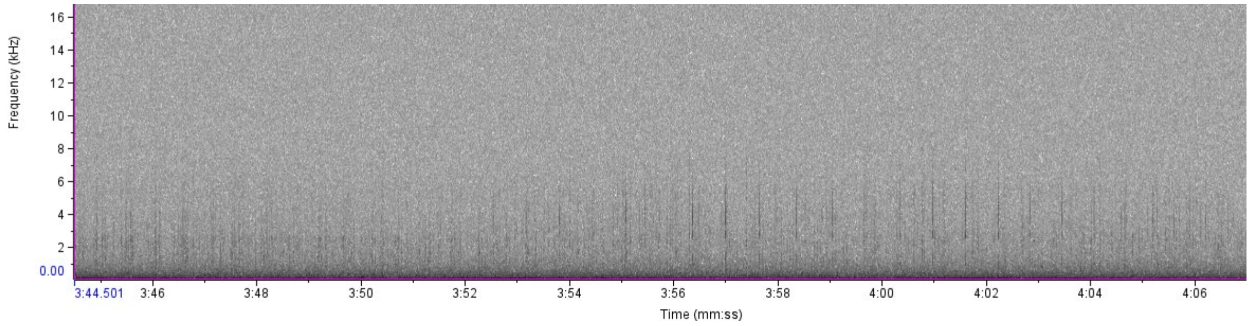

Figure C 16: Spectrogram showing 'faint' sperm whale clicks (1024 FFT size, 50% overlap, Hann window) missed by the automated detector.

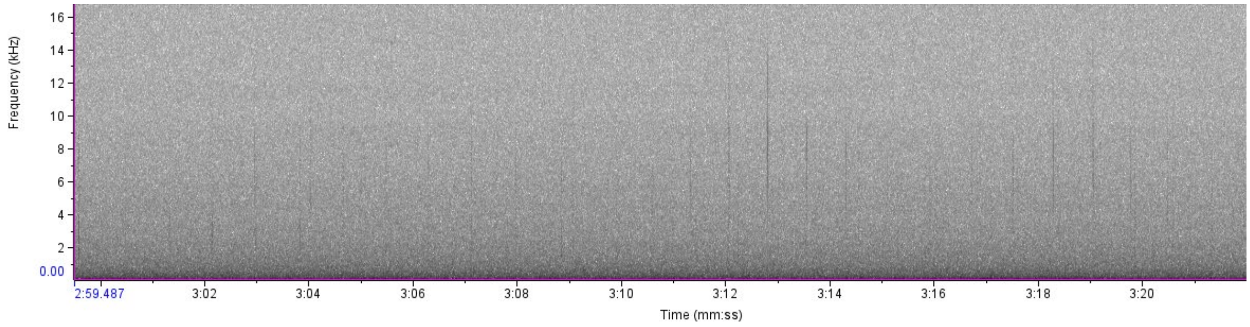

Figure C 17: Spectrogram showing 'faint' sperm whale clicks (1024 FFT size, 50% overlap, Hann window) missed by the automated detector.

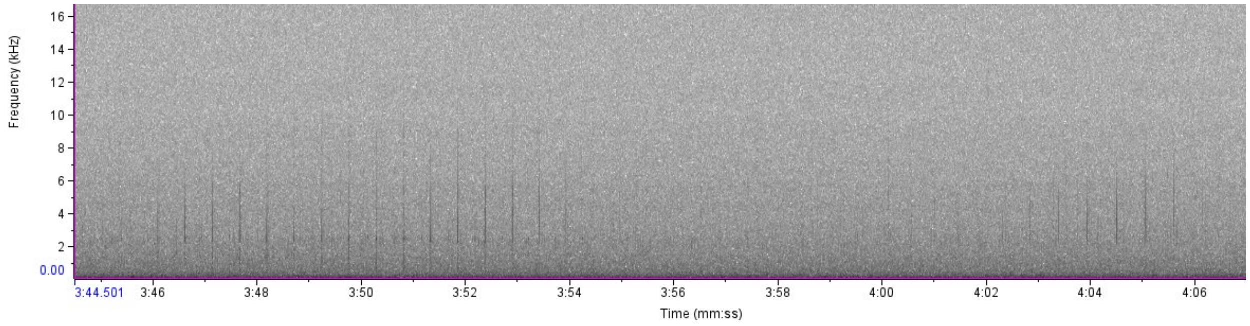

Figure C 18: Spectrogram showing 'faint' sperm whale clicks (1024 FFT size, 50% overlap, Hann window) missed by the automated detector.
